# Shared biophysical mechanisms determine early biofilm architecture development across different bacterial species

**DOI:** 10.1371/journal.pbio.3001846

**Published:** 2022-10-26

**Authors:** Hannah Jeckel, Francisco Díaz-Pascual, Dominic J. Skinner, Boya Song, Eva Jiménez-Siebert, Kerstin Strenger, Eric Jelli, Sanika Vaidya, Jörn Dunkel, Knut Drescher

**Affiliations:** 1 Biozentrum, University of Basel, Basel, Switzerland; 2 Department of Physics, Philipps-Universität Marburg, Marburg, Germany; 3 Max Planck Institute for Terrestrial Microbiology, Marburg, Germany; 4 Department of Mathematics, Massachusetts Institute of Technology, Cambridge, Massachusetts, United States of America; 5 Max Planck Institute for Neurobiology of Behavior, Bonn, Germany; Hebrew University, ISRAEL

## Abstract

Bacterial biofilms are among the most abundant multicellular structures on Earth and play essential roles in a wide range of ecological, medical, and industrial processes. However, general principles that govern the emergence of biofilm architecture across different species remain unknown. Here, we combine experiments, simulations, and statistical analysis to identify shared biophysical mechanisms that determine early biofilm architecture development at the single-cell level, for the species *Vibrio cholerae*, *Escherichia coli*, *Salmonella enterica*, and *Pseudomonas aeruginosa* grown as microcolonies in flow chambers. Our data-driven analysis reveals that despite the many molecular differences between these species, the biofilm architecture differences can be described by only 2 control parameters: cellular aspect ratio and cell density. Further experiments using single-species mutants for which the cell aspect ratio and the cell density are systematically varied, and mechanistic simulations show that tuning these 2 control parameters reproduces biofilm architectures of different species. Altogether, our results show that biofilm microcolony architecture is determined by mechanical cell–cell interactions, which are conserved across different species.

## Abstract 2

Bacterial biofilms are highly abundant forms of microbial life. Using single-cell-resolution structural images of biofilms comprising several different bacterial species, this study employs a data-driven approach to identify fundamental biophysical principles of biofilm development, conserved across species.

## Introduction

Bacterial biofilms are multicellular communities that grow on surfaces within a self-produced extracellular matrix [1,2]. Major research efforts over the past 2 decades [3–7] have established the ecological, biomedical, and industrial importance of bacterial biofilms and revealed that biofilms are highly abundant on Earth [8]. They are formed by many different species, in a multitude of different environments on many different types of interfaces. This diversity is reflected in the resulting biofilm architectures, which range from microscopic cell aggregates to macroscopic colonies, and to thick mats of cells that cover surfaces [1,5,8]. Biofilm architecture is impacted by a variety of external and internal cues including the nutritional environment [9,10], shear flow [11–13], motility and quorum sensing properties of the biofilm-forming strain [9,14,15], as well as the composition and properties of the extracellular matrix, which varies widely between different species [7,16–18].

Despite the molecular dissimilarities, biofilms of different species generally share a robustness against mechanical and chemical perturbations, and it is not well understood how these multicellular properties of biofilms arise from the collective growth and spatiotemporal self-organization of the communities. Recent advances in live imaging techniques make it possible to observe the development of early-stage biofilms at single-cell resolution, starting from a single founder cell up to a few thousand cells [13,19–22]. Imaging-based studies have provided key insights into the importance of mechanical cell interactions [21,23–30], cell surface attachment [25,31–35], growth memory [22], external fluid flow [13,36,37], and the external mechanical environment [38–40] for the emergent architecture in biofilms. However, these studies were restricted to a single species and it remains an open question whether there are common biophysical principles that govern biofilm architecture development across species.

To tackle this problem, we report here a combined experimental and theoretical investigation of three-dimensional (3D) biofilm architectures for the bacterial species, *Vibrio cholerae*, *Escherichia coli*, *Salmonella enterica*, and *Pseudomonas aeruginosa*, which are grown in microfluidic flow chambers as microcolonies up to a cell number of approximately 2,000 cells. Each of these species displays different growth characteristics, extracellular matrix components, cell morphology, and biofilm architectures. To identify common architectural characteristics across different bacterial species and to ultimately identify conserved biophysical principles for biofilm development, it is necessary to have quantitative metrics enabling comparisons between multicellular structures, which are able to robustly distinguish different biofilm architectures. Building on recent tools for 3D biofilm image analysis [41], we are able to extract and quantify numerous single-cell properties and emergent collective properties from microscopy image data of individual biofilms. To analyze these measurements, we introduce here a statistical metric framework based on a general Chebyshev representation of the experimentally measured parameter distributions, which is able to distinguish different biofilm species based on their architectural features. This metric overcomes limitations of previous methods that relied on the assumption of normally distributed data [21]. Since the underlying mathematical formulations of our analysis framework of 3D multicellular structures is generic, the method will be broadly applicable to other prokaryotic and eukaryotic multicellular structures in the future.

Through the quantitative biophysical analysis methodology outlined above, we find that emergent architectural differences across biofilms of different species correlate with variations in cell shape and local cell density. To test whether these correlations are due to causal relationships, we used mutants of a single species and particle-based computational modeling to independently explore the biophysical phase space of early-stage biofilm architectures. These experiments and simulations showed that 2 mechanical parameters (cell aspect ratio and the cell–cell attraction) jointly determine the emergent biofilm architecture across different species, which reveals a conserved principle for architecture development of biofilm microcolonies.

## Results and discussion

### Quantifying early-stage biofilm architecture across species

**Fig 1:**
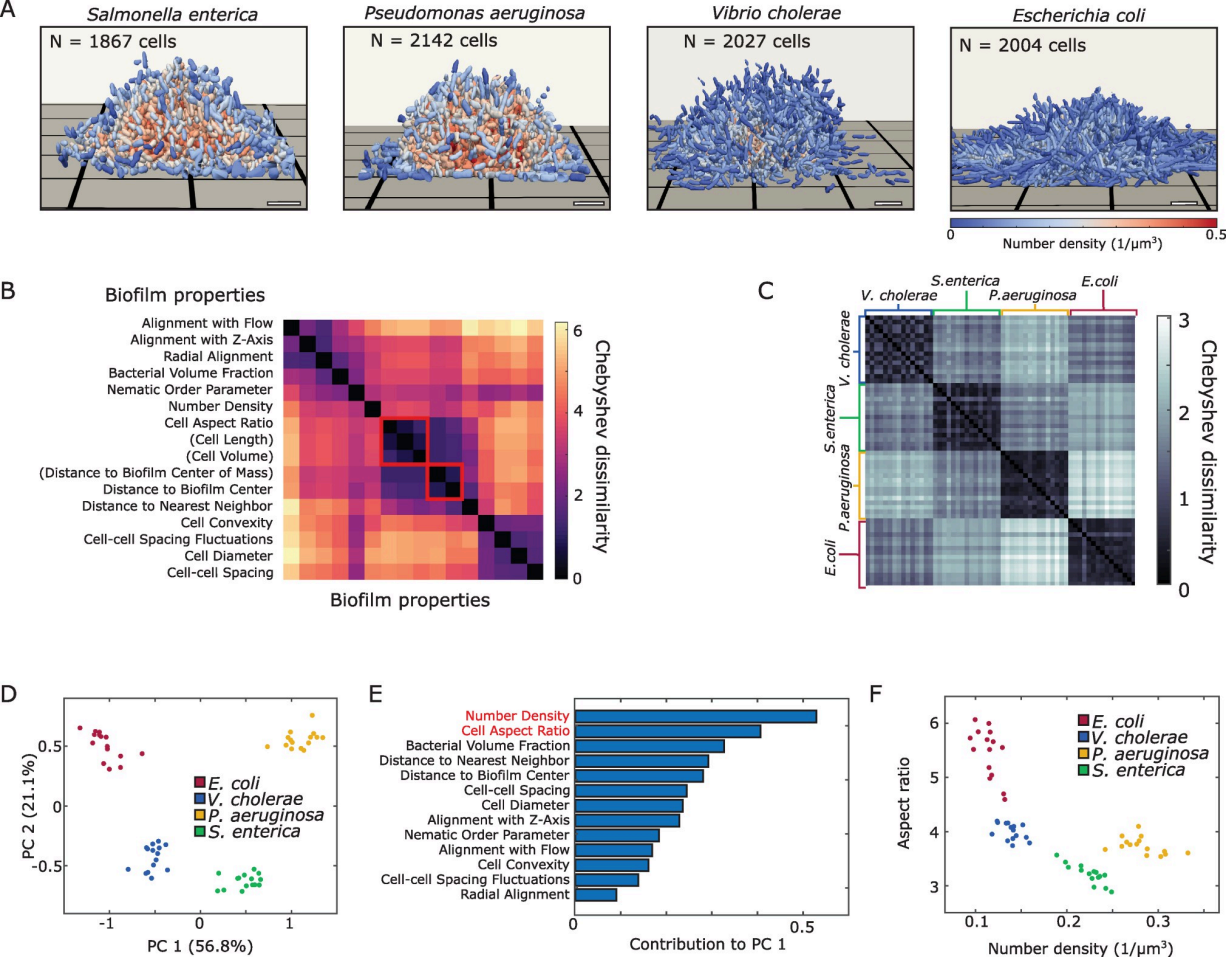
Early-stage biofilm architectures of different bacterial species can be quantitatively distinguished within a two-dimensional phase diagram derived from a statistical analysis of architectural properties. (**A**) Representative 3D biofilm microcolony architectures of 4 bacterial species reconstructed from segmented confocal microscopy images at comparable cell numbers (approximately 2,000 cells). Each cell is colored according to the local density within its neighborhood, of radius 2 μm. Scale bars, 5 μm. (**B**) For each biofilm, we approximated the distributions of 16 measured properties with Chebyshev polynomials (Section C in S1 Text). Using the Chebyshev polynomials for each measured property, a Cd measure is defined (Section C in S1 Text). Using this measure, highly correlated properties are identified and reduced as indicated by red squares, leaving *p* = 13 relevant properties. (**C**) The Cd also provides a robust and quantitative comparison of biofilm architectures from different species, as indicated by the block structure in this diagram. (**D**) PCA based on the Chebyshev coefficient space (Section C in S1 Text) robustly distinguishes biofilms of the 4 different species, *E*. *coli*, *V*. *cholerae*, *P*. *aeruginosa*, and *S*. *enterica*. (**E**) Cell aspect ratio and local number of neighbors are the key contributors to the first principal component (Section C, Fig D in S1 Text). (**F**) Representing the experimental data in the mean aspect ratio vs. cell number density plane confirms that these 2 properties define a biophysically interpretable phase diagram to categorize biofilm architectures. Source data are available at DOI: 10.5281/zenodo.7077624. Cd, Chebyshev dissimilarity; PCA, principal component analysis; 3D, three-dimensional.

To investigate the structural differences between biofilm architectures within and across bacterial species, we performed single-cell resolution imaging. For each of the 4 species, *E*. *coli*, *V*. *cholerae*, *P*. *aeruginosa*, and *S*. *enterica*, 15 biofilms were grown in microfluidic flow chambers from a few surface-attached founder cells until they reached around 2,000 cells, followed by imaging using confocal microscopy (Fig 1A; Materials and methods). For these biofilm sizes, the cells are expected to grow exponentially throughout the microcolonies [21]. Although all species formed colonies, the biofilm architectures of the 4 species were qualitatively different (Fig 1A). To quantify the observed differences in biofilm shape and structure between species, we segmented all individual cells in all biofilms following [21]. Using the software tool BiofilmQ [41], we measured for each biofilm several single-cell properties such as cell length, cell diameter, and cell convexity, together with emergent collective properties, such as local cell number density and nematic order, resulting in a histogram for every one of the *m* = 16 measured properties (see complete list in Table D, Section B in S1 Text). Each biofilm is thus represented by a set of *m* histograms.

Previous approaches have used mean- and variance-based measures of these histograms [21] to distinguish biofilm architecture; however, these measures do not carry information about the histogram’s shape and are therefore of limited utility. To broaden the scope of our statistical analysis and, therefore, the range of systems that it can be applied to, we sought a more general approach to systematically compare sets of histograms. To this end, we represented each empirically measured histogram with a Chebyshev polynomial of degree *d* = 20 using kernel density estimation (Section C, Fig A in S1 Text). Replacing approximately 2,000 single-cell measurements for each biofilm and each parameter with *d* + 1 = 21 polynomial coefficients allowed us to compress the experimentally observed data while retaining information about their distributions beyond mean values and variances. From a (*d* + 1) × *m* matrix containing all the Chebyshev coefficients for a given biofilm, we constructed a Chebyshev dissimilarity (Cd) measure, to compare 2 such matrices and, hence, 2 biofilms (Section C in S1 Text). Mathematically, Cd provides an upper bound on the cumulative *L*_1_-distance between collections of histograms. Similarly, taking a vector of Chebyshev coefficients constructed from a single property across all biofilms allows us to apply Cd to compare similarities of measured properties (Section C in S1 Text). Some properties, such as the cellular aspect ratio (the ratio of cell length to cell width) and cell length, can be expected to be closely related to each other and therefore add redundant information to the analysis. To prevent double-counting, we identified these highly correlated properties by performing clustering based on Cd and using the silhouette coefficient to determine the optimal cluster number (more details are provided in Section C in S1 Text). This analysis left us with *p* = 13 essential properties, which characterize biofilm architecture (Fig 1B). When calculating Cd for each pair of biofilms using the 13 essential properties, we observe a robust distinction according to species, as evident from the block structure in Fig 1C.

### Data-driven identification of the phase diagram of early-stage biofilm architecture

Principal component analysis (PCA) applied to the flattened (*d* + 1) × *p* = 21 × 13 dimensional vectors of Chebyshev coefficients representing each biofilm revealed that there are 4 distinct clusters corresponding to the 4 bacterial species (Fig 1D). The information contained in the *p* = 13 distributions of measured parameters is therefore sufficient to capture the key architectural differences between species.

The first principal component, which explains more than 50% of the variation in the data, can be used as a scalar measure for biofilm architecture and will from here on be referred to as the biofilm architecture index (BAI). To investigate which of the measured properties could be responsible for the interspecies variation, we examined the contributions of each parameter to the BAI (Fig 1E). The feature that contributed most to the BAI is the local cell number density, defined as the number of neighbors that a cell has within a 2-μm radius. The second highest contributing feature was the cell aspect ratio. The prominent contributions of the cell number density and cell aspect ratio to the BAI suggest that variations in these 2 parameters across biofilms could be responsible for variation in the observed architectures. To verify that these 2 properties provide the basis for a suitable biophysical phase diagram of biofilm architecture, we plot each biofilm in the mean cell number density versus mean cell aspect ratio plane (Fig 1F). The clear separation of the 4 species in this two-dimensional phase space shows that biofilm architectures can be efficiently characterized by these 2 parameters. We note that classical liquid crystals can also be characterized by an aspect ratio versus number density phase diagram [42,43], which highlights an interesting analogy between passive nematic structures and growth-active nematic biofilms.

### Altering biofilm architecture with cell aspect ratio mutants and cell–cell adhesion mutants

**Fig 2:**
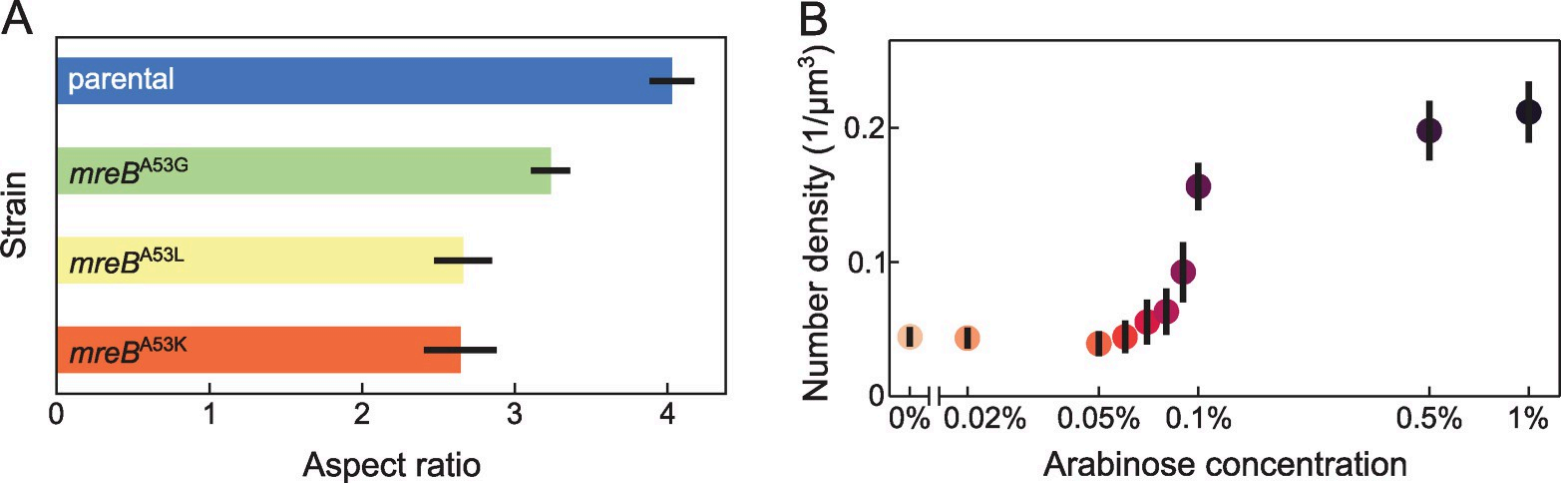
Cell aspect ratio and cell–cell adhesion can be precisely controlled in mutant strains. (**A**) Using different *V*. *cholerae* strains, the cell aspect ratio can be experimentally varied. Measurements were performed for *n* = 30 biofilms, including strains with point mutations in the *mreB* gene. Bar height corresponds to means; error bars indicate standard deviations. (**B**) Through additional mutations, the local number density can be varied experimentally, independent from the cell aspect ratio. Biofilms grown in the presence of different arabinose concentrations (*n* = 12 biofilms for each arabinose concentration) exhibit an increasing local number density with increasing arabinose concentration. Measurements were performed and averaged for Δ*rbmA* strains with either the wild-type *mreB*, *mreB*^A53K^, *mreB*^A53L^, or *mreB*^A53G^, harboring a plasmid with an arabinose-inducible *rbmA* expression construct (P_*BAD*_-*rbmA*). Data points are colored according to the arabinose concentration; error bars indicate standard deviations. Source data are available at DOI: 10.5281/zenodo.7077624.

The 4 species analyzed in Fig 1 differ in a large number of biological properties beyond the cell aspect ratio and number density. To test if cell aspect ratio and local density not only correlate with but also determine the different biofilm architectures observed across the 4 species, we generated several mutants in a single species, *V*. *cholerae*. By analyzing the biofilm architectures that arise from mutants within a single species, it is possible to isolate the effects of cell aspect ratio and local density on the biofilm architecture. To this end, we generated mutations in *mreB*, following [44], which resulted in different aspect ratios compared to the parental strain (Fig 2A). For altering the cell aspect ratio, we preferred using *mreB* mutations instead of using antibiotics (such as cephalexin), because these mutations did not interfere with bacterial replication rates as shown in Fig E in S1 Text.

**Fig 3:**
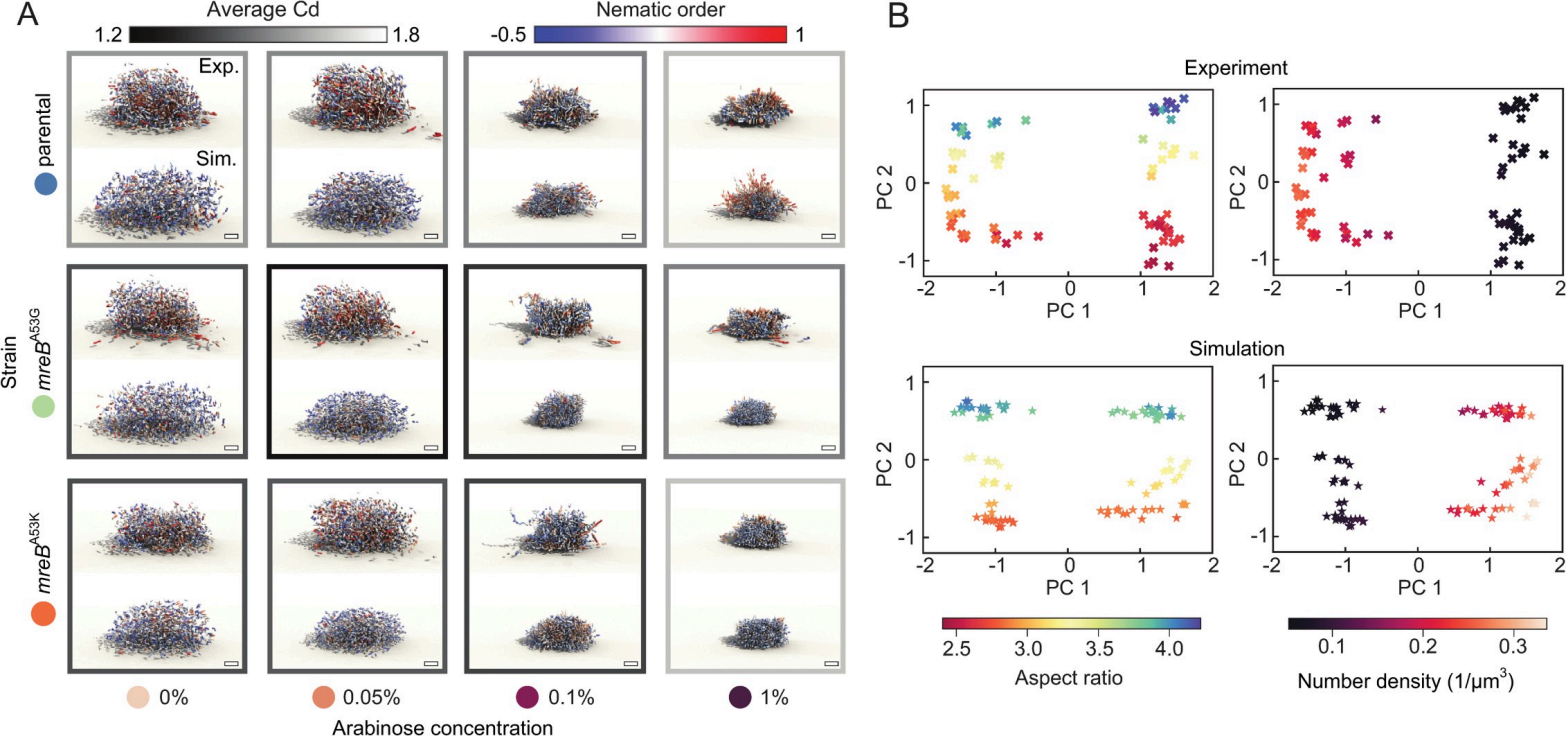
Experiments and simulations show that differences in biofilm architecture are driven by cell aspect ratio and cell–cell adhesion. (**A**) Renderings of *V*. *cholerae* biofilms (top, *N*_cell_ approximately 2,000) and corresponding best-fit simulations (bottom, *N*_cell_ = 2,000) for combinations of 3 different mutants (arranged vertically) and 4 different arabinose concentration levels (arranged horizontally). Each cell in the biofilm is colored by the local nematic order around it. The outline of each grid panel is colored in grayscale by the average Cd between the corresponding experiments (*n* = 3) and the best-fit simulation (see Fig J in S1 Text for the exact values). Scale bar, 5 μm. (**B**) Two-dimensional PCA embedding of the Chebyshev features of *n* = 72 *V*. *cholerae* mutant biofilms (top) and a group of *n* = 114 simulations consisting of the top 5 best-fitting simulations (bottom) for each combination of strain and arabinose concentration. The PCA embedding is colored by average aspect ratio (left) and average local number density (right) of all the cells in each biofilm, confirming that these 2 parameters are principal determinants of biofilm architecture, consistent with Fig 1E. Source data are available at DOI: 10.5281/zenodo.7077624. Cd, Chebyshev dissimilarity; PCA, principal component analysis.

To control the cell density, we introduced mutations that alter the abundance of the cell–cell attraction-mediating matrix protein RbmA [19,21]; specifically, we deleted the native *rbmA* gene from the chromosome and reintroduced a copy of *rbmA* under the control of a promoter that is inducible by the monosaccharide arabinose (Materials and methods). By growing the cells in the presence of different levels of arabinose, we can therefore tune the level of RbmA production (Section D, Fig G(b), Section E in S1 Text), which changes the cell–cell attraction, ultimately resulting in different cell number densities (Fig 2B, Section D and Fig H in S1 Text). The presence of arabinose in our glucose-based growth medium also has a small positive influence on the cellular growth rate (Fig F in S1 Text). We then introduced the *rbmA* mutation and inducible *rbmA* expression construct into the parental *V*. *cholerae* strain, as well as in strains with smaller aspect ratios (the *mreB* mutants). Using these strains, we then performed a comprehensive experimental scan over the different cell aspect ratios and cell densities, which resulted in widely different biofilm architectures (Fig 3A).

Control experiments revealed a small effect of the *mreB* mutations on both the level of RbmA produced (Fig G(b) in S1 Text) and on the biofilm density (Fig H in S1 Text). This effect of the *mreB* mutations, however, does not interfere with any conclusions drawn of the experimental scan over the different cell aspect ratios and cell densities because our conclusions are based on the cell density that was actually measured in the biofilms, independent of the factors that contributed to it.

To understand whether the natural phase diagram of biofilm architectures for the different *V*. *cholerae* mutants is, like the phase diagram for the different species introduced in (Fig 1F), also based on the cell aspect ratio and cell number density, we again performed PCA. Applying PCA to the vectors of Chebyshev coefficients for each biofilm and coloring the data points by aspect ratio (Fig 3B, top left) and number density (Fig 3B, top right) reveals that these parameters exactly correspond to the first 2 principal components of this embedding. Therefore, the appropriate phase diagram of biofilm architectures of *V*. *cholerae* mutants spanned by the aspect ratio and number density, consistent with the results for the different species in Fig 1F.

### Computational model for biofilm growth based on mechanical interactions reproduces experimental biofilm architectures

Cell aspect ratio and cell–cell attraction, which were systematically varied for *V*. *cholerae* experimentally (Fig 2A and 2B), are key parameters for the mechanical cell–cell interactions. To test if the effect of these parameters on the biofilm architecture is primarily due to changes in mechanical cell–cell interactions, we compared the experimental measurements for the *V*. *cholerae* strains with a computational model for biofilm growth in which cells only interact mechanically (Fig 3A). In this model, which extends a previously introduced simulation framework [13,21], individual cells are represented as growing, dividing ellipsoids that experience pairwise cell–cell interactions and cell–surface interactions that determine their overdamped positional and orientational dynamics. The cell–cell interactions account for both short-range steric repulsion together with RbmA-mediated attraction [13,21]. In addition to cell–surface steric repulsion [13,21], our simulations now also include an effective cell–surface attraction to account for the surface attachment of *V*. *cholerae* before and during biofilm formation [45,46]. To further refine the previously introduced minimal model [13,21], we implemented strongly anisotropic friction effects to account for the fact that the matrix polymer network can suppress the transverse motions of cells [47–49] (Section F in S1 Text). We generally found that the inclusion of the cell anchoring to the bottom surface and the anisotropic matrix-mediated friction leads to a substantially improved agreement between experimentally observed and simulated biofilms (Fig 3A), when comparing their architectural properties in terms of the Cd measure (Section F in S1 Text).

To compare the experimental biofilm architectures of the *V*. *cholerae* mutants with the computational model, we performed systematic parameter scans to identify the values of simulation parameters that correspond to a given experimental system. Specifically, we performed *>*6,000 simulations to search the parameter space of cell length at the time of division, range of cell–cell repulsion force, range of cell–cell attraction force, and strength of the cell–cell attraction (Section F in S1 Text), with the remaining parameters determined from a previous experimental biofilm calibration [13,21] (see Table E in S1 Text). The best-fitting parameter values for a given experiment were determined by taking the values with the smallest Cd between experiment and simulation (Fig I in S1 Text). Using the fitted parameter values, we see a qualitative agreement between the experiment and simulation across various combinations of cell aspect ratio mutants and arabinose concentration levels (Fig 3A). This agreement between the biofilm architectures obtained from the experimental parameter scan and the simulation parameter scan indicates that changes in cell aspect ratio and cell–cell attraction cause changes in the biofilm architecture through their effects on mechanical cell–cell interactions.

Analogous to our analysis of experimental biofilm data from *V*. *cholerae* mutants (Fig 3B, top row), we again applied PCA to the Chebyshev coefficients of *n* = 114 simulated biofilms and colored the points according to aspect ratio (Fig 3B, bottom left) and number density (Fig 3B, bottom right). Consistent with the experimental results, these diagrams reveal that the principal component axes correspond to the number density and aspect ratio, respectively. Similar to the results for the different species (Fig 1F) and the *V*. *cholerae* mutants (Fig 3B, top row), the PCA for the simulations (Fig 3B, bottom row) indicates that the appropriate phase diagram of biofilm architectures is spanned by the aspect ratio and number density.

### Biofilm architecture of one species can be transformed into architecture of another species by changing mechanical control parameters

**Fig 4:**
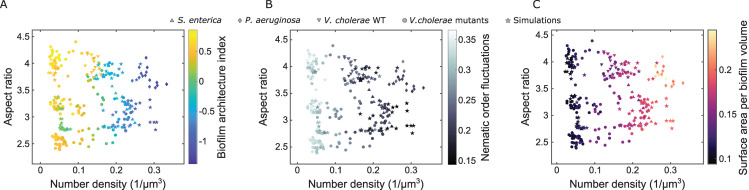
Joint phase diagram combining experimental biofilms from different species shown in Fig 1A with the experimental biofilms of *V*. *cholerae* mutants and the simulated biofilms from Fig 3. Each biofilm in the phase diagram is (**A**) colored by the biofilm architecture index BAI, (**B**) colored by an emergent collective property of the biofilm architecture, the variance of the nematic order parameter, and (**C**) colored by another emergent collective property, the surface area per biofilm volume. Source data are available at DOI: 10.5281/zenodo.7077624.

Given that the cell aspect ratio and number density in biofilms are the key control parameters for the biofilm architecture, we sought to understand which emergent architectural features change in the aspect ratio–density phase plane, and which conclusions can be drawn from these changes. We therefore plot the experimental biofilms for the different species and *V*. *cholerae* mutants together with our simulation results in the aspect ratio–density phase plane (Fig 4) and color-code different emergent properties of the biofilm architecture in each panel: Fig 4A shows the BAI, and Fig 4B and 4C show the nematic order fluctuations and the biofilm surface area per volume, respectively. The nematic order fluctuations and the biofilm surface area per volume are both independent from our statistical analysis, which ensures that our observations are not a particularity of the BAI but also reflected in other biofilm architecture–related measures. The graphs in Fig 4 show that number density is the key contributor to biofilm architecture, and cell aspect ratio has a more subtle influence.

The emergent properties of the biofilm architecture in Fig 4 for the experimental and simulated biofilms agree very well for all regions in the phase diagram, indicating that the mechanics-based simulations capture the emerging biofilm architecture, irrespective of the particular species under investigation. Even though the specific molecular structure and composition of the extracellular matrix differ widely for the different species, these molecular details only indirectly influence this phase diagram through the number density.

Finally, the phase diagrams in Fig 4 show that while the data points from each species inhabit a particular region in the phase plane, the *V*. *cholerae* mutants spread across the phase plane regions of different species. In each phase plane region, the emergent properties of the biofilm architecture of the *V*. *cholerae* mutants match those of the particular species inhabiting this phase plane region. These results show that the biofilm architecture of *V*. *cholerae* can be modified to reproduce the biofilm architecture of other species by simply tuning the control parameters of the phase diagram (cell aspect ratio and cell number density).

## Conclusions

By performing single-cell resolution imaging on early-stage bacterial biofilms of several bacterial species, we found that the emergent early biofilm architecture correlates with differences in cell aspect ratio and local cell number density. By systematically varying the aspect ratio and cell–cell attraction using mutants of a single bacterial species, we then showed that these parameters determine the observed architectural differences. Extensive particle-based simulations of biofilm growth support this conclusion and further revealed that the impact of these parameters on the emergent biofilm architecture reflects the underlying effective mechanical cell–cell interactions. Our combined experimental and theoretical results show that bacterial biofilm architectures populate an aspect ratio–number density phase diagram, similar to classical liquid crystals. By changing the cell aspect ratio and number density of a particular species, this species can reproduce biofilm architectures of other species, even though the extracellular matrix composition and cellular properties can differ widely between species. It remains unclear to which extent principles revealed in this study for the importance of mechanical cell–cell interactions in biofilm microcolonies also dictate the architecture development of larger biofilms, which can display significant metabolic heterogeneity and additional types of cell–cell interactions may become important.

## Materials and methods

### Bacterial strains and media

All *V*. *cholerae* strains used in this study are derivatives of a rugose variant of the O1 biovar El Tor wild-type strain N16961 [50]. The *E*. *coli* strain used in this study (KDE2011) is a derivative of the AR3110 wild type [51], carrying a point mutation in the promoter of the gene *csgD*, which increases biofilm formation [52]. The *S*. *enterica* strain used here (KDS38) is a derivative of the UMR1 wild type [53], carrying a mutation in the promoter of *csgD* (formerly called *agfD* in *Salmonella*), which increases biofilm formation [54]. The point mutations in the *E*. *coli* and *S*. *enterica* strains were necessary to grow isolated biofilm colonies in our experimental conditions. The *P*. *aeruginosa* strain used here (KDP63) is a derivative of the PAO1 wild type [55] (obtained from Urs Jenal, Basel). The *V*. *cholerae*, *E*. *coli*, and *S*. *enterica* strains carried a plasmid driving the production of sfGFP using the P_*tac*_ promoter. The *P*. *aeruginosa* strain KDP63 carried a high-copy number plasmid producing the fluorescent protein YPet under the control of a pX2 promoter [56].

To engineer *V*. *cholerae* strains with a different cell length and width, amino acid 53 of the native MreB protein was replaced according to Monds and colleagues [44]. These modifications were introduced to the chromosome of *V*. *cholerae* by conjugation using the *E*. *coli* strain S17-1 *λpir* [57] and the pKAS32 suicide vector [58], containing *mreB* with the corresponding mutation and 500 bp upstream and 500 bp downstream from the codon that codes for amino acid 53 of MreB. To control the expression of *rbmA* in *V*. *cholerae*, inducible strains were created by conjugating a plasmid that contained P_*tac*_*-sfGFP* and P_*BAD*_*-rbmA* constructs. This plasmid enabled us to vary the production of RbmA by adding different concentrations of arabinose to the growth medium [21]. All strains, plasmids, and oligonucleotides that were used in this study are listed in Tables A, B, and C in S1 Text, respectively.

For overnight cultures or strain construction, cells were either grown in liquid Luria–Bertani–Miller broth (LB-Miller; 10 g L^−1^ tryptone, 5 g L^−1^ yeast extract, and 10 g L^−1^ NaCl) or LB-Miller without salt (10 g L^−1^ tryptone and 5 g L^−1^ yeast extract) with the corresponding antibiotic and shaking at 250 rpm, or using agar-solidified LB media (containing 1.5% agar). All *V*. *cholerae* biofilm experiments were performed in M9 minimal medium, with the following composition: M9 minimal salts (M6030, Sigma), 2 mM MgSO_4_, 100 μM CaCl_2_, MEM vitamins, 0.5% glucose, 15 mM triethanolamine (pH 7.1), and gentamicin (30 μg mL^−1^). *E*. *coli* biofilm experiments were performed in tryptone broth (10 g L^−1^ tryptone) supplemented with kanamycin (50 μg mL^−1^). *S*. *enterica* biofilm experiments were performed in tryptone broth supplemented with spectinomycin (100 μg mL^−1^). *P*. *aeruginosa* biofilm experiments were performed in FAB medium, with the following composition: CaCl_2_ (11 mg L^−1^), MgCl_2_ (93 mg L^−1^), (NH_4_)_2_SO_4_ (2 g L^−1^), Na_2_HPO_4_·2H_2_O (6 g L^−1^), KH_2_PO_4_ (3 g L^−1^), NaCl (3 g L^−1^), glucose (25 ml L^−1^), and the trace metals solution (100 ml L^−1^). The trace metals solution consists of CaSO_4_·2H_2_O (2 mg L^−1^), FeSO_4_·7H_2_O (2 mg L^−1^), MnSO_4_·H_2_O (0.2 mg L^−1^), CuSO_4_·5H_2_O (0.2 mg L^−1^), ZnSO_4_·7H_2_O (0.2 mg L^−1^), CoSO_4_·7H_2_O (0.1 mg L^−1^), NaMoO_4_·H_2_O (0.1 mg L^−1^), and H_3_BO_3_ (0.05 mg L^−1^).

### Flow chamber biofilm experiments

Biofilms were grown in microfluidic flow chambers, which were made from polydimethylsiloxane bonded to glass coverslips using an oxygen plasma, with 4 to 8 identical flow channels on a single coverslip. All flow rates were controlled using a syringe pump (PicoPlus, Harvard Apparatus). The microfluidic channels were 500 μm wide and 7 mm long. For *V*. *cholerae*, *E*. *coli*, and *S*. *enterica*, channels with height 100 μm were used, whereas for *P*. *aeruginosa*, channels with height 300 μm were used. Each biofilm is considered as a biological replicate.

For *V*. *cholerae* biofilm growth, overnight cultures grown in liquid LB-Miller with gentamicin (30 μg mL^−1^) at 28°C were diluted 1:200 into fresh LB-Miller with gentamicin and grown for 2 h. Then, these cultures were adjusted to an optical density at 600 nm (OD_600_) of 0.001 and used to inoculate a microfluidic channel. The cells were given 1 h at room temperature to attach to the glass surface without flow, before fresh M9 medium with gentamicin was flown through the channel at a rate of 50 μL min^−1^ for 45 s to wash away the nonattached cells. Then, the flow rate was set to 0.5 μL min^−1^ for the remainder of the experiment, and the flow channel as incubated at 25°C.

For *E*. *coli* biofilm growth, overnight cultures were grown in liquid LB-Miller with kanamycin (50 μg mL^−1^) at 37°C. These cultures were diluted 1:2,000 into tryptone broth and used to inoculate a microfluidic flow chamber. The cells were given 1 h to attach to the substrate without flow, before washing away nonadherent cells using tryptone broth with kanamycin at a flow rate of 50 μL min^−1^ for 45 s. Then, the flow rate was set to 0.1 μL min^−1^ for the remainder of the experiment, and the flow channel was incubated at 25°C.

For *S*. *enterica* biofilm growth, overnight cultures were grown at 37°C in liquid LB-Miller without salt, supplemented with spectinomycin (100 μg mL^−1^). The overnight cultures were diluted 1:2,000 and used to inoculate a flow channel. After giving the cells 1 h to attach to the coverslip without flow, the nonattached cells were washed away with tryptone broth supplemented with spectinomycin for 45 s using a flow rate of 50 μL min^−1^. The flow rate was then set to 0.1 μL min^−1^ for the remainder of the experiment, and the flow channel was incubated at 25°C.

*P*. *aeruginosa* strains were grown overnight in 5 ml liquid LB-Miller with 30 μg mL^−1^ gentamicin at 37°C with shaking. The overnight culture was back-diluted 1:200 in 3 mL LB-Miller and grown until OD_600_ = 0.5. This culture was subsequently diluted 1:1,000 in FAB medium and used to inoculate microfluidic flow chambers. After allowing cells to attach to the glass coverslip for 1 h at 30°C without flow, the cells were washed for 50 s using a flow rate of 200 μL min^−1^. The flow rate was then set to 3 μL min^−1^ for the remainder of the experiment, and the flow channel was incubated at 30°C.

### Image acquisition

Biofilms were imaged using an electron-multiplying charge-coupled device camera (EMCCD, iXon, Andor) and a Yokogawa confocal spinning disk unit mounted on a Nikon Ti-E inverted microscope, and an Olympus 100× silicone oil (refractive index = 1.406) objective with a 1.35 numerical aperture. The fluorescent protein sfGFP was excited using a 488-nm laser. Three-dimensional images were acquired during biofilm growth every 60 min, using a *z*-spacing of 400 nm. The hardware was controlled using Matlab (MathWorks). A live feedback between image acquisition, image analysis, and microscope control was used to automatically detect the biofilm and expand the imaging field during growth in 3D, as described by Hartmann and colleagues [21], to minimize the laser exposure of the growing biofilm. Image analysis methods are described in detail in the S1 Text Section B.

## Supporting information

S1 Text File containing supplementary Tables A–E and supplementary Figs A–J and additional description of image analysis methods, Chebyshev dissimilarity, individual-based model and simulations, properties of mutant strains, and proteomics methods. (PDF)

## Abbreviations

BAI: biofilm architecture index
Cd: Chebyshev dissimilarity
LB-Miller: Luria–Bertani–Miller
PCA: principal component analysis
3D: three-dimensional
