# Supplementary material for: Shared biophysical mechanisms determine early biofilm architecture development across different bacterial species
Source: PLoS Biol. 2022 Oct 26;20(10):e3001846. doi: 10.1371/journal.pbio.3001846 (PMC9605341; doi:10.1371/journal.pbio.3001846)
Supplement: S1 Text — (PDF) [file pbio.3001846.s001.pdf]

Supplementary information:
Shared biophysical mechanisms determine early biofilm architecture development across different bacterial species

Hannah Jeckel,[1,2,*] Francisco Díaz-Pascual,[3,*] Dominic J. Skinner,[4,*] Boya Song,[4,*] Eva Jiménez-Siebert,[1] Kerstin Strenger,[1] Eric Jelli,[5] Sanika Vaidya,[3] Jörn Dunkel,[4,†] and Knut Drescher[1,†]

[1] *Biozentrum, University of Basel, Spitalstrasse 41, 4056 Basel, Switzerland*
[2] *Department of Physics, Philipps-Universität Marburg, Karl-von-Frisch-Str. 16, 35043 Marburg, Germany*
[3] *Max Planck Institute for Terrestrial Microbiology, Karl-von-Frisch-Str. 16, 35043 Marburg, Germany*
[4] *Department of Mathematics, Massachusetts Institute of Technology, 77 Massachusetts Avenue, Cambridge, MA 01239, USA*
[5] *Max Planck Institute for Neurobiology of Behavior, Ludwig-Erhard-Allee 2, 53175 Bonn, Germany*

CONTENTS

A. Strains, plasmids, oligonucleotides 2

B. Image analysis methods 4
Adaptations to segmentation algorithm from Ref. [1] 4
Quantification of biofilm architecture 4
Single-cell properties 4
Collective multicellular properties 5
Characterization of biofilms in terms of histogram of measured properties 5
Kernel smoothing of histogram 5

C. Chebyshev dissimilarity 8
Spectral approximation of probability distributions 8
Chebyshev dissimilarity 8
Comparing two biofilms by the statistical properties 8
Comparing two properties across a collection of biofilms 9
Avoiding over-weighting similar properties 10
Contributions of each property to the principal components 10

D. Properties of mutant strains 12
Impact of *mreB* mutations on biofilm growth rate 12
Impact of arabinose concentration on biofilm growth rate 12
Influence of arabinose concentration and *mreB* mutations on RbmA production 13
Influence of arabinose concentration and *mreB* mutations on cell density in biofilms 13

E. Proteomics methods 15
Sample Preparation 15
LC-MS Analysis 15

F. Individual-based model and simulations 16
Model description 16
Model implementation 17
Simulation parameters 17

References 20

* These authors contributed equally
† To whom correspondence should be addressed. E-mail: dunkel@mit.edu, knut.drescher@unibas.ch

A. STRAINS, PLASMIDS, OLIGONUCLEOTIDES

Table A: Bacterial strains used in this study.

Strain	Genotype, relevant features	Reference
Vibrio cholerae		
KDV101	Wild type strain N16961 (O1 El Tor)	Meibom *et al.* [2]
KDV148	N16961, $vpvC^{\mathrm{W240R}}$ (matrix hyperproducer phenotype)	Drescher *et al.* [3]
KDV611	N16961, $vpvC^{\mathrm{W240R}}$ $\Delta crvA$	Hartmann *et al.* [1]
KDV613	N16961, $vpvC^{\mathrm{W240R}}$ $\Delta crvA$, contains plasmid pNUT542	Hartmann *et al.* [1]
KDV615	N16961, $vpvC^{\mathrm{W240R}}$, contains plasmid pNUT542	Hartmann *et al.* [1]
KDV1082	N16961, $vpvC^{\mathrm{W240R}}$, $\Delta crvA$, $\Delta rbmA$, contains plasmid pNUT1519	Hartmann *et al.* [1]
KDV1232	N16961, $vpvC^{\mathrm{W240R}}$, $\Delta crvA$, $mreB^{\mathrm{A53K}}$. Codon "AAA" used to encode lysine	This study, MreB point mutation according to Ref. [4]
KDV1248	N16961, $vpvC^{\mathrm{W240R}}$, $\Delta crvA$, $mreB^{\mathrm{A53K}}$, contains plasmid pNUT542	This study
KDV1291	N16961, $vpvC^{\mathrm{W240R}}$, $\Delta crvA$, $mreB^{\mathrm{A53L}}$. Codon "CTG" used to encode leucine	This study, MreB point mutation according to Ref. [4]
KDV1295	N16961, $vpvC^{\mathrm{W240R}}$, $\Delta crvA$, $mreB^{\mathrm{A53L}}$, contains plasmid pNUT542	This study
KDV1403	N16961, $vpvC^{\mathrm{W240R}}$, $\Delta crvA$, $mreB^{\mathrm{A53G}}$. Codon "GGT" used to encode glycine	This study, MreB point mutation according to Ref. [4]
KDV1404	N16961, $vpvC^{\mathrm{W240R}}$, $\Delta crvA$, $mreB^{\mathrm{A53G}}$, contains plasmid pNUT542	This study
KDV1688	N16961, $vpvC^{\mathrm{W240R}}$, $\Delta crvA$, $mreB^{\mathrm{A53K}}$, $\Delta rbmA$	This study
KDV1690	N16961, $vpvC^{\mathrm{W240R}}$, $\Delta crvA$, $mreB^{\mathrm{A53L}}$, $\Delta rbmA$	This study
KDV1692	N16961, $vpvC^{\mathrm{W240R}}$, $\Delta crvA$, $mreB^{\mathrm{A53G}}$, $\Delta rbmA$	This study
KDV1694	N16961, $vpvC^{\mathrm{W240R}}$, $\Delta crvA$, $mreB^{\mathrm{A53K}}$, $\Delta rbmA$, contains plasmid pNUT1519	This study
KDV1696	N16961, $vpvC^{\mathrm{W240R}}$, $\Delta crvA$, $mreB^{\mathrm{A53L}}$, $\Delta rbmA$, contains plasmid pNUT1519	This study
KDV1698	N16961, $vpvC^{\mathrm{W240R}}$, $\Delta crvA$, $mreB^{\mathrm{A53G}}$, $\Delta rbmA$, contains plasmid pNUT1519	This study
Escherichia coli		
KDE474	*E. coli* AR3110 wild type	Serra *et al.* [5]
KDE671	AR3110, point mutation inside *csgD* promoter according to Grantcharova *et al.* [6]	Vidakovic *et al.* [7]
KDE2011	KDE671 strain carrying plasmid pNUT1361.	This study
S17-1 λpir	$\Delta lacU169$ ($\phi lacZ\Delta$M15), *recA*1, *endA*1, *hsdR*17, *thi*-1, *gyrA*96, *relA*1, λpir	de Lorenzo *et al.* [8]
TOP10	*mcrA*, Δ(*mrr-hsdRMS-mcrBC*), ϕ80*lacZ*(*del*)M15, $\Delta lacX74$, *deoR*, *recA*1, *araD*139, Δ(*ara-leu*)7697, *galU*, *galK*, *rpsL*(SmR), *endA*1, *nupG*	Invitrogen
Salmonella enterica		
MAE52	UMR1 $\mathrm{P}_{agfD1}/\mathrm{rdar}_{28/37}$	Römling *et al.* [9]
KDS38	MAE52, contains plasmid pNUT541 plasmid	Drescher lab stock
Pseudomonas aeruginosa		
UJP505	*Pseudomonas aeruginosa* PAO1 wild type	Malone *et al.* [10]
KDP63	UJP505, contains plasmid pNUT1636	This study

Table B: Plasmids used in this study. Abbreviations: Kan = kanamycin, Amp = ampicillin, Gent = gentamicin, Spect = spectinomycin. Superscript "R" indicates resistance, and "-" indicates a fusion.

**Plasmid**	**Origin, marker, and relevant features**	**Reference**
pKAS32	pR6K, Amp^R	Skorupski and Taylor [11]
pNUT144	pR6K, Amp^R, Kan^R	Drescher *et al.* [12]
pNUT336	pR6K, Amp^R, with 1 kb upstream and downstream region of *rbmA* to create Δ*rbmA* strains	Nadell *et al.* [13]
pNUT541	pSC101*, Spect^R, P$_{tac}$-*sfgfp-sfgfp*	Drescher lab stock
pNUT542	pSC101*, Gent^R, P$_{tac}$-*sfgfp-sfgfp*	Singh *et al.* [14]
pNUT1519	pSC101*, Gent^R, P$_{tac}$-*sfgfp-sfgfp*, P$_{BAD}$-*rbmA*	Hartmann *et al.* [1]
pNUT1636	pBBR1, Gent^R, *pX2-ypet*	Drescher lab stock
pNUT1361	pSC101*, Kan^R, P$_{tac}$-*sfgfp-sfgfp*	Drescher lab stock
pNUT1711	pR6K, Amp^R, Kan^R, contains 500 bp upstream and 500 bp downstream of codon for amino acid 53 in *mreB*	This study
pNUT1727	pR6K, Amp^R, Kan^R, contains *mreB*^{A53K} mutation and 500 bp upstream and 500 bp downstream from that region	This study
pNUT1745	pR6K, Amp^R, Kan^R, contains *mreB*^{A53L} mutation and 500 bp upstream and 500 bp downstream from that region	This study
pNUT1744	pR6K, Amp^R, Kan^R, contains *mreB*^{A53G} mutation and 500 bp upstream and 500 bp downstream from that region	This study

Table C: DNA oligonucleotides used in this study.

**Name**	**Sequence (5' to 3' direction)**	**Description**
KDO2144	CGGAATATTAATGGAGCAGGGGCTCTTGGTGT	pNUT1711 construction
KDO2147	ATGCATCCTAGGCCTTTGATCTTTTCTGCAGTGGCTTCAC	pNUT1711 construction
KDO2145	AGCCCCTGCTCCATTAATATTCCGGAGTATACGTAGCCGG	pNUT1711 construction
KDO2146	TGCAGAAAAGATCAAAGGCCTAGGATGCATATGGCG	pNUT1711 construction
KDO1879	AACGGTTTTACCGCCACGACCTTTG	pNUT1727, pNUT1744 and pNUT1745 construction
KDO1878	AAAGCGGTTGGCCATGCCGCAA	pNUT1727 construction
KDO2179	GGTGCGGTTGGCCATGCCGCAA	pNUT1744 construction
KDO2178	CTGGCGGTTGGCCATGCCGCAA	pNUT1745 construction

B. IMAGE ANALYSIS METHODS

Adaptations to segmentation algorithm from Ref. [1]

The segmentation algorithm defined in Ref. [1] is optimized for *V. cholerae* bacteria, whose aspect ratio is typically around 2-3. For longer cells, such as *E. coli* bacteria with an aspect ratio up to 5, oversegmentation was observed when applying this standard algorithm, making adjustments necessary. To avoid such oversegmentation, we reduced the number of watershed seeds prior to applying the watershed algorithm, greatly improving the accuracy of *E.coli* segmentation.

In order to identify watershed seeds to remove, we first determine which watershed seeds lie within the same bacterial cell. We achieve this by using the filtered intensity image to identify whether connecting lines between pairs of seeds experience a drop in intensity. This is to be expected when the seeds lie within different cells. In contrast, if there is no intensity drop along the connecting line, it is likely that the seeds are located within the same cell.

For a pair of seeds p_a and p_b we first calculate the pixels p_i that represent the connecting line between them. By convention we set $p_0 = p_a$ and $p_N = p_b$, where N is the number of pixels representing the line. Denoting the value of the filtered intensity image at pixel p_i as I_i, we define the expected intensity E_i at p_i to be

$$E_i = I_a + \frac{i}{N} \cdot (I_b - I_a) \tag{S1}$$

and the deviation D_i of from this expectation as

$$D_i = (I_i - E_i)/E_i \,. \tag{S2}$$

With this definition, a large negative value for D_i corresponds to a significant intensity drop between the seeds, indicating that they are located in different cells. If any of the pixels p_i correspond to background, the deviation D_i is set to $-\inf$. The intensity drop $d_{a,b}$ for the pair of seeds p_a and p_b is defined as $\min(D_i)$.

After determining $d_{a,b}$ for each pair of seeds with a distance of 100 pixels (63µm) or less between them, a cutoff c is then calculated by applying Otsu's method to the distribution of all finite values of $d_{a,b}$. All pairs of seeds above this cutoff value are considered to belong to the same cell. After clustering all seeds located in the same cell and choosing only one representative seed for each cell, the watershed algorithm and all subsequent steps are applied as described in [1].

Quantification of biofilm architecture

To quantify the architecture of biofilms, cells were segmented using the algorithm described in [1], with modifications as described above. This segmentation was then imported into BiofilmQ [15] and used for subsequent analysis.

For each biofilms, we measured both single-cell properties (cell size, length, aspect ratio, etc.), and collective multicellular properties (local cell density, nematic order, etc.); where by definition, the computation of collective multicellular properties requires input information from two or more cells, whereas the single-cell properties can be determined by only knowing information about a single cell. All measured properties are listed in Table D. All properties were calculated with BiofilmQ, some of which were calculated using standard BiofilmQ functionality and some of which required the use of a custom BiofilmQ user-defined parameter script. We have indicated properties where a custom script was required in Table D, and for these properties, we explain their definition in detail below.

Single-cell properties

To obtain the cell size parameters and orientations, an ellipsoidal fit was applied to each segmented cell. This fit yielded a vector for each of the three axes of symmetry. The length and orientation of the longest axis of symmetry is assigned to be the cell length and cell orientation respectively, and the second longest axis defines the cell diameter. The alignment with the z-axis and the direction of flow is defined to be π minus the relative angle between those axes and the cell orientation obtained by the ellipsoidal fit. To calculate the radial alignment, the vector pointing from cell to the biofilm center of mass projected to the substrate is used as a reference, π minus the relative angle between this vector and the cell's orientation defines the radial alignment.

Collective multicellular properties

Let $\mathcal{S}_\alpha(r)$ be the sphere of radius r around cell α, and $\mathcal{N}_\alpha(r)$ the set of other cells with centroids in $\mathcal{S}_\alpha(r)$. The local density at cell α is defined to be the volume fraction occupied by cells in $\mathcal{S}_\alpha(r)$. The local number density is defined to be number of cells in $\mathcal{N}_\alpha(r)$ divided by the volume of $\mathcal{S}_\alpha(r)$. For this work, we chose $r = 2$µm to determine the local density.

To determine the nematic order parameter, we set $r = 3$µm, such that about 5-8 cells can be expected to be within the sphere $\mathcal{S}_\alpha(r)$. Let $\hat{\boldsymbol{n}}_\alpha$ be the normalized orientation vector for the cell α and for each $\gamma \in \mathcal{N}_\alpha(r)$, let $\hat{\boldsymbol{n}}_\gamma$ be the corresponding normalized orientation vector. Then, the nematic order parameter of the cell α is defined as

$$NO_\alpha = \left[\frac{1}{|\mathcal{N}_\alpha|}\sum_{\gamma\in\mathcal{N}_\alpha}\frac{3}{2}(\hat{\boldsymbol{n}}_\alpha \cdot \hat{\boldsymbol{n}}_\gamma)^2\right] - \frac{1}{2} \tag{S3}$$

For calculating the cell-cell spacing, the outline of each cell is approximated by a triangulated mesh using the MATLAB isosurface implementation. Along the direction of the normal vector of each face of the mesh, the distance to the next object surface is determined. The cell-cell spacing is given by the mean of these distances, the cell-cell spacing fluctuations by the standard deviation of them.

Characterization of biofilms in terms of histogram of measured properties

Each biofilm is characterized by a collection of histograms summarizing the single-cell properties (centroid coordinates, cell length, aspect ratio, cell orientation, etc.) and collective cell properties (local density, local nematic order, etc.). A quantitative comparison of two biofilms can thus be performed by comparing their sets of corresponding histograms. Below, we describe a systematic procedure for comparing two biofilms based on spectral Chebyshev approximations of the measured histograms.

Kernel smoothing of histogram

To reconstruct the probability density function from the histograms, we first remove the outliers of the measurements (if any) for each property and each biofilm, defined as measurements that are 4 standard deviations away from the mean. We then compute the range of each biofilm property across a set of biofilms by finding the minimum and the maximum of the measurements from all biofilms, and normalize those measurements so that they lie between -1 and 1. This normalization step allows us to combine biofilm properties with different units when comparing biofilms. For each property and each biofilm, we use kernel density estimation to fit the underlying probability density function by applying a Gaussian kernel with a standard deviation of 0.08 to the normalized measurements. Fig A shows the histograms of the normalized measurements, and the resulting kernel density estimates of the probability density function for all biofilm properties.

Parameter	Parameter name in BiofilmQ	Parameter description	Unit
**Cell aspect ratio**	Custom parameter (Ellipsoidal fit)	Ratio of longest axis to second-longest axis from ellipsoidal fit	None
**Cell convexity**	Shape_Convexity	Ratio of cell volume to convex cell volume	None
**Cell diameter**	Custom parameter (Ellipsoidal fit)	Second longest axis length of ellipsoidal fit	µm
**Cell length**	Custom parameter (Ellipsoidal fit)	Longest axis length of ellipsoidal fit	µm
**Cell volume**	Shape_Volume	Volume of the object	μm^3
**Alignment with flow**	Custom parameter (Ellipsoidal fit)	Angle between direction of flow and ellipsoidal longest axis direction	Radian
**Alignment with *z*-axis**	Custom parameter (Ellipsoidal fit)	Angle between z-axis and ellipsoidal longest axis direction	Radian
**Radial alignment**	Custom parameter (Ellipsoidal fit)	Angle between vector pointing to center of biofilm at substrate and ellipsoidal direction	Radian
**Nematic order parameter**	Custom parameter	Nematic order parameter based on all neighbors found within a sphere of radius 3 µm around the cell's centroid	None
**Bacterial volume fraction**	Architecture_Local Density_range32	Occupied volume fraction in a sphere of radius 2 µm around the cell centroid	None
**Number density**	Architecture_Local Number Density_range32	Number density of cell centroids present in a sphere of radius 2 µm around the object (including itself)	None
**Distance to biofilm center at substrate**	Distance_ToBiofilm CenterAtSubstrate	Distance of the cell to the center of mass of the biofilm projected onto the substrate	µm
**Distance to biofilm center**	Distance_ToBiofilm CenterOfMass	Distance of the cell to the center of mass of the biofilm	µm
**Distance to nearest neighbor**	Distance_ToNearest Object_ch1	Centroid-centroid distance of the cell to the closest different cell	µm
**Cell-cell spacing**	Custom parameter	Distance from the cell's surface to the closest surface of another cell in a defined direction, averaged over directions across the cell body given by an isosurface approximation	µm
**Cell-cell spacing fluctuations**	Custom parameter	Standard deviation of the distance between cell surfaces as described for cell-cell spacing across all directions used	µm

Table D: Summary of biofilm properties measured for architecture quantification. Single cell properties are listed first and separated from collective multicellular properties by a double line. Measurements based on custom code are described in more detail. All other properties were calculated using BiofilmQ version 0.1.6, available at https://drescherlab.org/data/biofilmQ/docs/usage/installation.html.

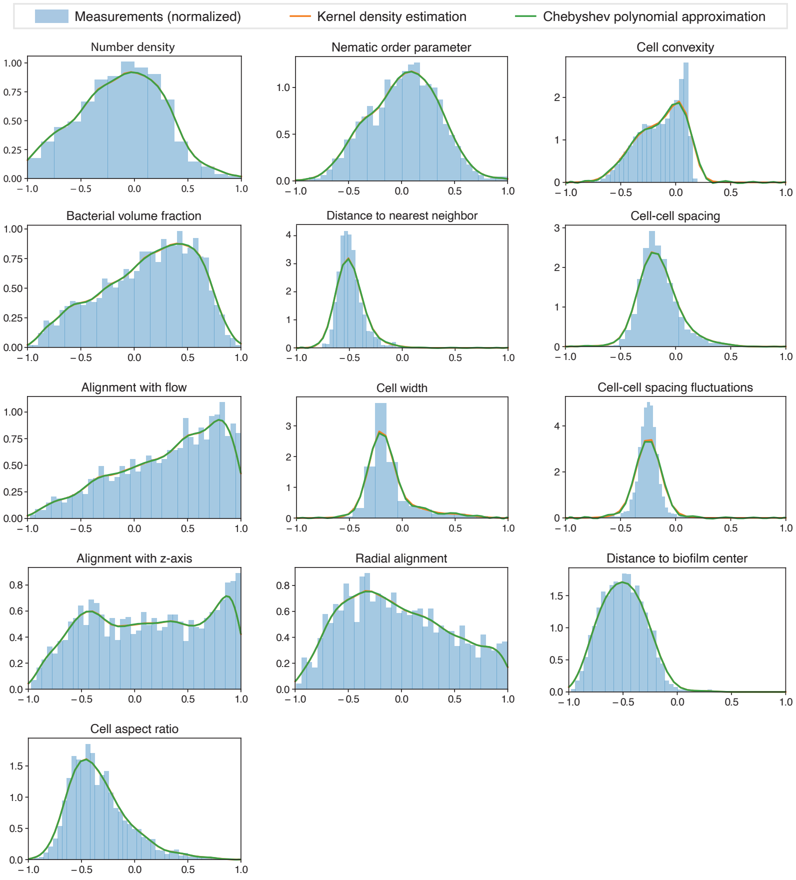

Fig A: Kernel density estimation and Chebyshev polynomial approximation for all the properties of a 2,300-cell *V. cholerae* biofilm (parental strain, 1% arabinose concentration), using the Chebyshev quantification method. Our kernel density estimations (orange lines) capture the overall shape of distributions, and are well-approximated by the Chebyshev expansion of degree 20 (green lines). Source data is available at DOI: 10.5281/zenodo.7077624.

C. CHEBYSHEV DISSIMILARITY

Here we introduce a method that allows us to distinguish and classify biofilms both within a species and across species based on the Chebyshev representation of the kernel density estimate.

Spectral approximation of probability distributions

One can approximate a continuous normalized probability density $f(x) \geq 0$ on $[-1, 1]$ by its Chebyshev expansion

$$F_d(x) = \sum_{k=0}^{d} f_k T_k(x) \tag{S4}$$

where $T_k(x) = \cos(k \arccos x)$ denotes the Chebyshev polynomials of the first kind.

To compute the Chebyshev features for a set of biofilms, we fit a Chebyshev polynomial with degree $d = 20$ to the kernel-smoothed probability density function to obtain 21 Chebyshev coefficients for each measured property of a biofilm. Fig A shows the resulting Chebyshev polynomial approximations for all the properties for a biofilm.

Chebyshev dissimilarity

Let $F_d(x) = \sum_{k=0}^{d} f_k T_k(x)$ and $G_d(x) = \sum_{k=0}^{d} g_k T_k(x)$ be two Chebyshev expansions of degree d that approximate functions $f(x)$ and $g(x)$ on the interval $x \in [-1, 1]$. The Chebyshev dissimilarity between $f(x)$ and $g(x)$ is defined by

$$\mathrm{Cd}[f, g] := \sum_{k=0}^{d} w_k \, |f_k - g_k| \,, \qquad w_k = \int_{-1}^{1} dx \, |T_k(x)| \leq 2. \tag{S5}$$

Cd gives an upper bound of the L_1-norm between $F_d(x)$ and $G_d(x)$, because

$$\begin{aligned} \int_{-1}^{1} dx \left| \sum_{k=0}^{d} (f_k - g_k) T_k(x) \right| &\leq \int_{-1}^{1} dx \sum_{k=0}^{d} |(f_k - g_k) T_k(x)| \\ &= \int_{-1}^{1} dx \sum_{k=0}^{d} |f_k - g_k| \, |T_k(x)| \\ &= \sum_{k=0}^{d} |f_k - g_k| \int_{-1}^{1} dx \, |T_k(x)| = \mathrm{Cd}[f, g] \end{aligned} \tag{S6}$$

In practice, we choose to compute Cd as an approximation of the L_1-norm between $F_d(x)$ and $G_d(x)$, as Cd can be evaluated much more efficiently. With this measure, modes are approximately equally weighted since $1 \leq w_k \leq 2$.

Comparing two biofilms by the statistical properties

Denote the probability density function of a property p in biofilm b as $f^{p,b}$. The Chebyshev dissimilarity between two biofilms b_1 and b_2 is computed as the average of the Chebyshev dissimilarities of all $p = 1, \ldots, P$ measured properties between two biofilms,

$$\mathrm{Cd}^{b_1, b_2} = \frac{1}{P} \sum_{p=1}^{P} \mathrm{Cd}[f^{p, b_1}, \, f^{p, b_2}]. \tag{S7}$$

Using this method, we computed the pairwise Cd between biofilms of four different species (panel B in Fig 1 in the main text), between *V. cholerae* biofilms (Fig B) and between *V. cholerae* biofilms and simulations to fit undetermined simulations parameters, as described in **Simulation parameters**.

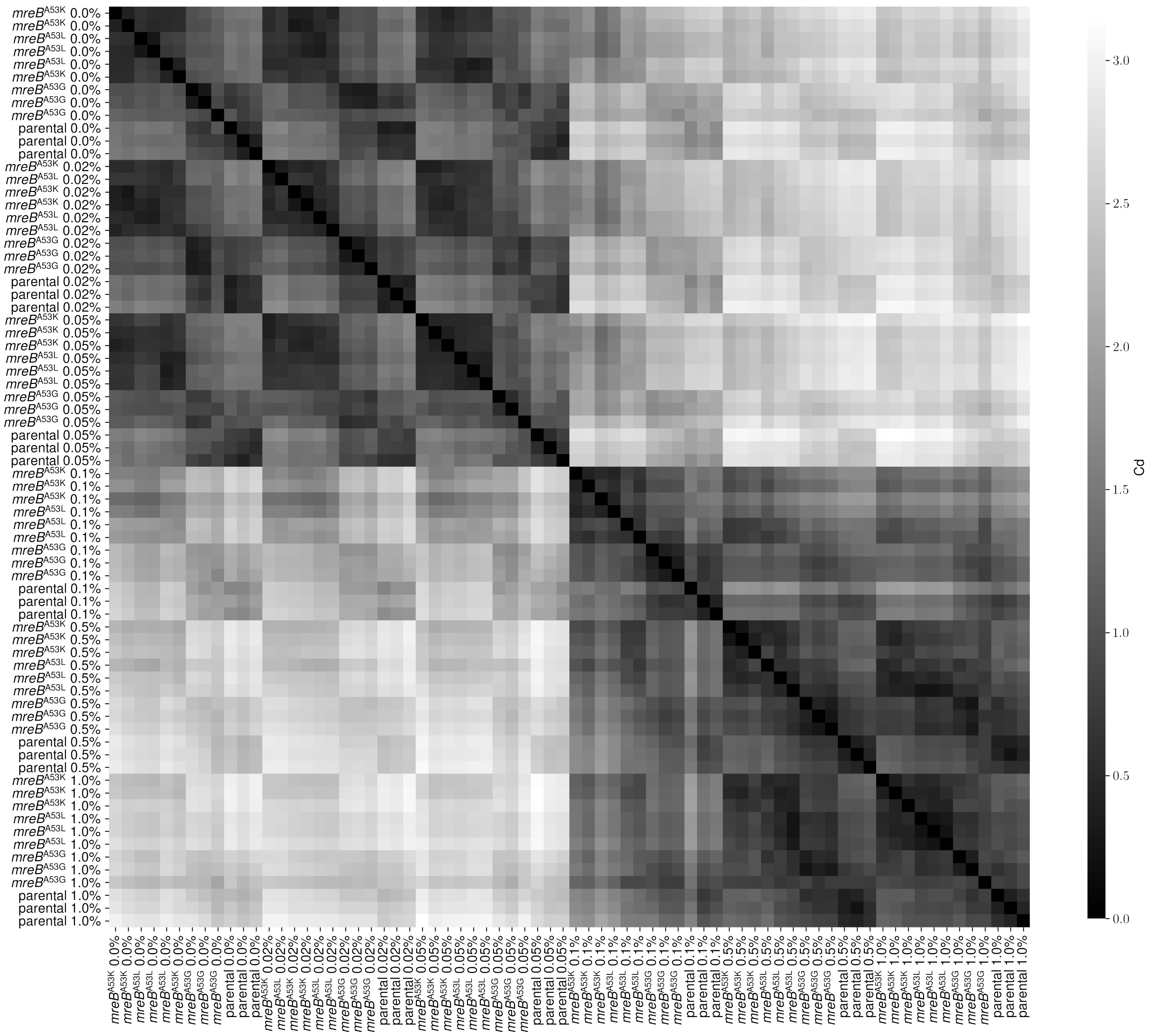

Fig B: Pairwise Chebyshev dissimilarity for 72 *V. cholerae* biofilms ($N_{\text{cell}} \sim 2000$) grown under different experimental conditions. The labels on x and y axis indicate the strain and the arabinose concentration level of each biofilm. Source data is available at DOI: 10.5281/zenodo.7077624.

Comparing two properties across a collection of biofilms

The Cd between two measured properties p_1 and p_2 is computed as the average of the Chebyshev dissimilarities between p_1 and p_2 in all $b = 1, \ldots, B$ biofilms,

$$\text{Cd}^{p_1,p_2} = \frac{1}{B} \sum_{b=1}^{B} \text{Cd}[f^{p_1,b}, f^{p_2,b}]. \tag{S8}$$

We computed the pairwise between all 16 measured properties of biofilms of four different species (Fig C and in panel C in Fig 1 in the main text) to identify highly-correlated clusters of properties, as described below.

Avoiding over-weighting similar properties

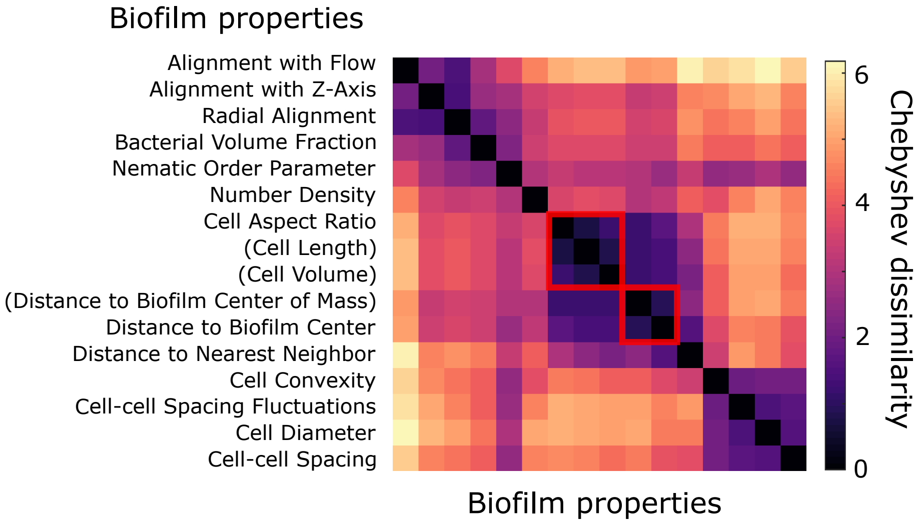

Fig C: Pairwise Cd between 16 properties measured in biofilms of four different species (15 biofilms each for *E. coli*, *V. cholerae*, *P. aeruginosa* and *S. enterica*; $N_{\text{cell}} \sim 2000$ for all the biofilms). Using silhuoette coefficient and hierarchical clustering, the optimal number of properties was identified to be 13. Clusters are highlighted by red squares. Source data is available at DOI: 10.5281/zenodo.7077624.

To avoid double counting highly-correlated biofilm properties in our comparison between two biofilms, we calculate pairwise Cd between all 16 measured properties of biofilms of four different species (15 biofilms each for *E. coli*, *V. cholerae*, *P. aeruginosa* and *S. enterica*; $N_{\text{cell}} \sim 2000$ for all the biofilms) and identify highly-correlated clusters of properties using hierarchical clustering. For each number of clusters, starting with 1 up until the total number of properties, we calculate the silhuoette coefficient for the clustering based on the hierarchical tree and found that the optimum is achieved at 13 clusters. As depicted in Fig C, which is also shown in panel B in main Fig 1, two clusters containing two and three properties respectively are identified and collapsed into single property measurements by choosing a representative.

Contributions of each property to the principal components

When applying a principal component analysis to the Chebyshev coefficient space, all four species can be distinguished clearly in the projection of the first two principal components, as shown in main text Fig 1. The contribution of each biofilm property to these components can be estimated by calculating the norm of the weight vector of the Chebyshev coefficients associated with the biofilm property. Fig D shows this contribution estimate for each biofilm property to the principal components 1 and 2, revealing that the aspect ratio as well as cell density in the form of number density and cell-cell spacing have a strong impact on the PCA results and therefore the biofilm architecture.

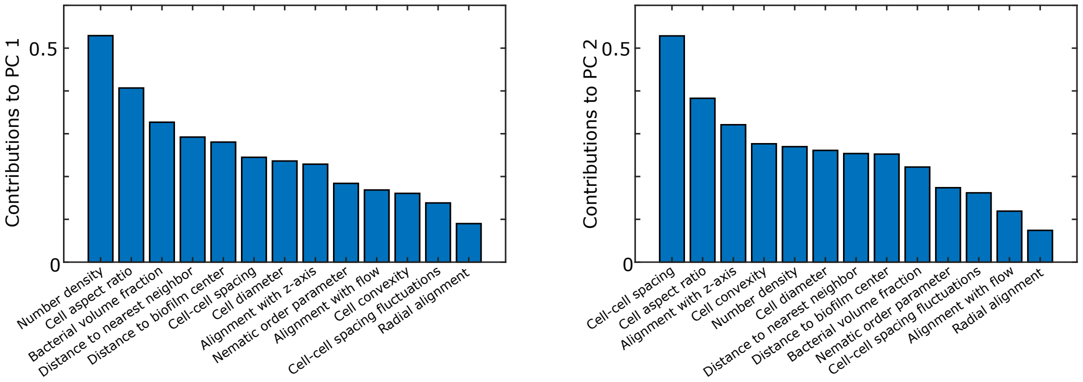

Fig D: The aspect ratio appears within the top two contributions for both PC1 and PC2, indicating that it plays in important role in biofilm architecture. Properties related to the cell density such as number density and cell-cell spacing also rank highly, which implies that the cell density also has a great impact on architecture. Source data is available at DOI: 10.5281/zenodo.7077624.

D. PROPERTIES OF MUTANT STRAINS

To systematically explore the effect that aspect ratio and local number density have on the biofilm architecture, mutants of *Vibrio cholerae* were constructed. For changing the aspect ratio of bacterial cells, *mreB* point mutations were introduced according to Ref. [4], leading to cell shapes with smaller aspect ratios as shown in the main text panel A in Fig 2. In each of those mutants as well as in the parental strains, additional mutations were introduced to allow for controlling the production of RbmA through addition of arabinose to the medium (see main text and material and methods). All strains, plasmids, and oligonucleotides that were used in this study are listed in Table A, Table B, and Table C, respectively.

Impact of *mreB* mutations on biofilm growth rate

To measure the growth rate of *mreB* mutants in biofilms, all single cells were detected in 3D confocal microscopy image time series of biofilm growth up to 2000 cells, using BiofilmQ [15] and the semgentation technique from Ref. [1]. We observed exponential growth under these conditions and extracted the growth rate as the inverse cell doubling time, excluding the lag phase from the calculation. Fig E shows the growth rates of each of the *mreB* mutants as well as the parental strain. While a general trend towards higher growth rates for lower aspect ratios can be observed, the differences are not statistically significant.

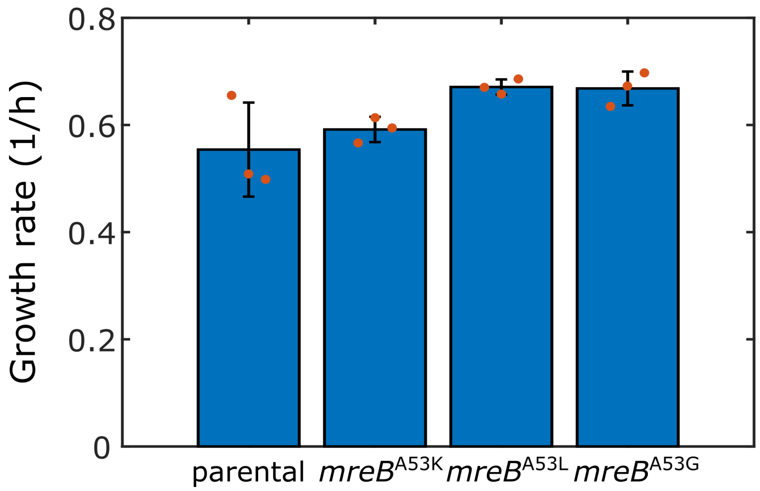

Fig E: The *mreB* mutations used in this study have no significant impact on cell doubling time in biofilms. Growth rates, defined as the inverse of the cell doubling time, are plotted for the parental strain and the three different *mreB* mutants. Source data is available at DOI: 10.5281/zenodo.7077624.

Impact of arabinose concentration on biofilm growth rate

In our experiments, *V. cholerae* biofilms are grown in M9 minimal medium with 0.5% glucose. For experiments involving strains that harbor a plasmid with the arabinose-inducible *rbmA* expression system P_{BAD}-*rbmA*, we measured the growth rate in biofilms as a function of arabinose concentration, because arabinose could potentially be used as a carbon source, even though glucose is a preferred carbon source. Fig F shows the growth rate of each biofilm as a function of arabinose concentration with individual data points colored by strain and bars and error bars representing the mean and standard deviation of all biofilms at a given arabinose concentration. A significant increase in growth rates between low and high arabinose concentrations can be observed, increasing the growth rate from ≈0.47/h to 0.67/h. It is therefore reasonable to assume that arabinose is in part used as a carbon source during biofilm growth in our conditions. However, since this project focused on end-point measurements at a fixed cell number, comparability of the data is still ensured, even when growth rates between individual biofilms differ.

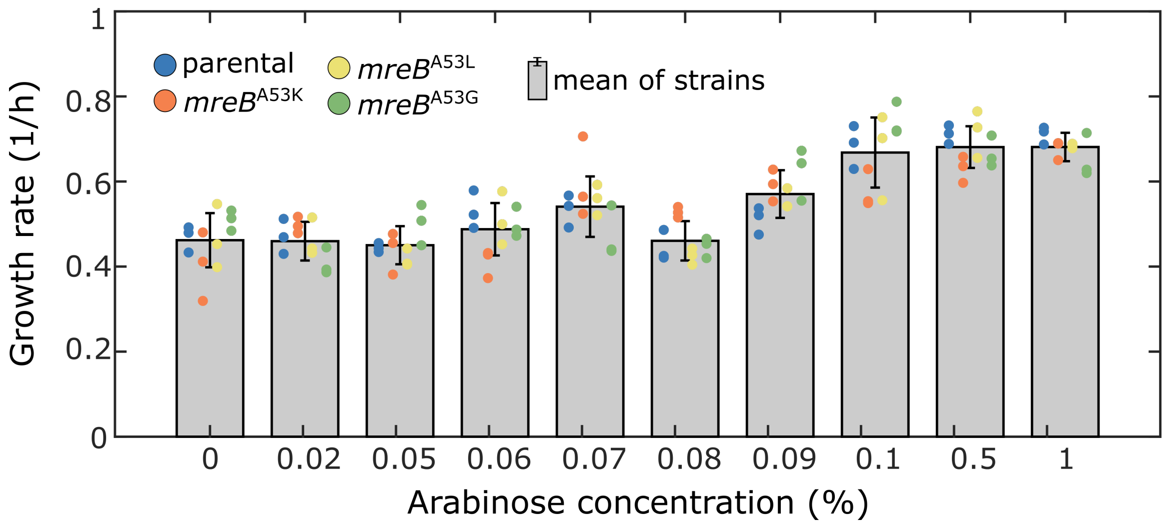

Fig F: High arabinose concentrations increase growth rates of *V. cholerae* biofilms in M9 medium with 0.5% glucose. Growth rates, defined as the inverse of the cell doubling time in the biofilm, are plotted for each biofilm as a function of different arabinose concentrations added to the medium. Grey bars and error bars represent mean and standard deviation of all biofilms grown at a given arabinose concentration. Data points represent data for individual biofilms, colored by strain. Source data is available at DOI: 10.5281/zenodo.7077624.

Influence of arabinose concentration and *mreB* mutations on RbmA production

First, we wanted to test whether the *mreB* mutations have an impact on RbmA levels. We therefore measured the fold-change of extracellular RbmA between each of the three *mreB* mutants and the parental strain, all harboring the $\Delta rbmA$ mutation and the P_{BAD}-*rbmA* construct, at a constant arabinose concentration of 0.08%. RbmA levels were determined by measuring the extracellular proteome using mass spectrometry. The results, shown in panel a in Fig G, indicate that there are small but significant differences in extracellular RbmA levels between the parental strain and the strains with *mreB* mutation A53K and A53L.

In strains carrying the $\Delta rbmA$ mutation and the P_{BAD}-*rbmA* construct, growth in increased arabinose concentrations should lead to increased RbmA levels. To test whether this is indeed observed in our conditions, we measured the extracellular proteome using mass spectrometry (media: M9 + 0.5%glucose + variable arabinose concentration). The proteome measurements were used to determine the extracellular RbmA levels. This experiment was performed for arabinose concentrations ranging from 0% (control) to 0.5%. Comparing the RbmA levels of the induced strains to the base level of RbmA production at 0% arabinose, shown in panel b in Fig G, reveals a significant increase of RbmA levels with increasing arabinose concentration.

Overall, these results indicate, that the P_{BAD}-*rbmA* construct has the expected influence on RbmA production, and that the *mreB* mutations have a small but significant impact on RbmA production.

Influence of arabinose concentration and *mreB* mutations on cell density in biofilms

Fig 2 of the main text shows the local number density as a function of arabinose concentration, displaying the mean and standard deviation, where data was pooled across the parental strain and the three *mreB* mutants. Different aspect ratios, however, may lead to differences in cell packing and therefore cell density, and the *mreB* mutations also have a slight impact on RbmA production as described above. It is therefore useful to disentangle the pooled mean from Fig 2 to ensure that it is justified to combine all mutants into a single average. Fig H shows the same graph as Fig 2, where in addition to the mean and standard deviation of all strains, depicted by grey bars and error bars respectively, the values for each biofilm are shown as points with the color corresponding to the strain. For low ($\leq 0.05\%$) and high ($\geq 0.1\%$) arabinose concentrations, there are no differences in the cell density between strains. For the intermediate concentrations 0.07%, 0.08% and 0.09% however, a trend towards higher cell number densities for smaller cell aspect ratios can be observed. In this intermediate range, number densities are also the most sensitive to small changes in arabinose concentrations, indicating that the biofilm architecture at this RbmA level is particularly sensitive to pertubations. The magnitude of cell density changes between different species is at a similar level as the

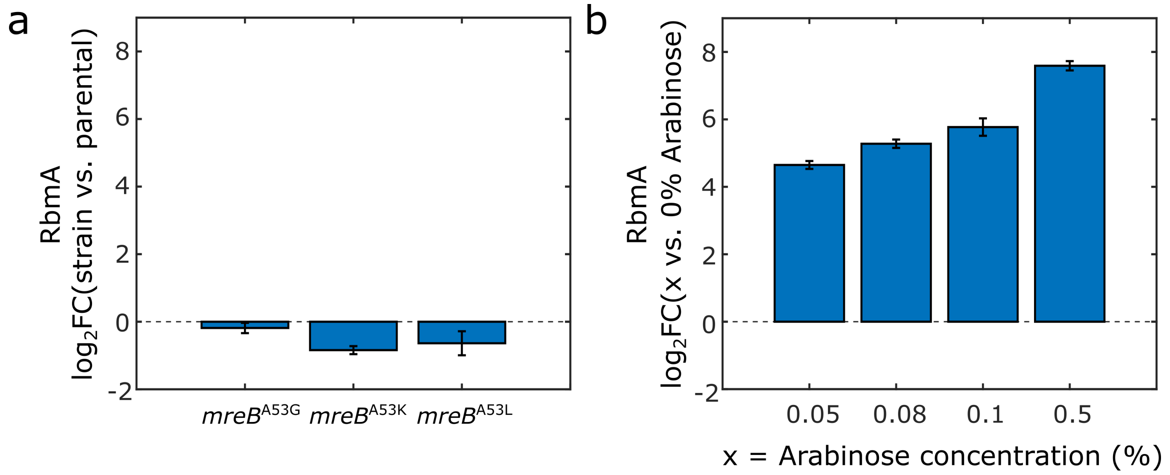

Fig G: RbmA level measurements in biofilms using mass spectrometry, for different *mreB* nutants and for biofilms grown in different arabinose concentrations. (a) Fold changes (FC) of extracellular RbmA levels between the *mreB* mutant strains and the parental strain, revealing a small, but significant effect for two of the three mutations. (b) Fold changes of extracellular RbmA levels of the strain KDV1082 ($\Delta rbmA$, P_{BAD}-$rbmA$) for different arabinose concentrations, relative to arabinose-free medium. For this strain, a higher concentration of arabinose results in higher levels of RbmA in the biofilms. All bar heights are calculated based on $n = 3$ independent biological replicates, error bars indicate standard deviation. Source data is available at DOI: 10.5281/zenodo.7077624.

magnitude of changes due to small ($< 0.02\%$) differences in arabinose concentration, but significantly lower than the magnitude of change due to large differences in arabinose concentrations. Overall, the experimental system is therefore suited to explore the density - aspect ratio space in a systematic manner, because we do not require cell densities of different mutants to be perfectly identical for identical arabinose concentrations, but rather rely on meaningful changes to occur across the variation of arabinose concentrations in order to observe different combinations of aspect ratio and cell densities.

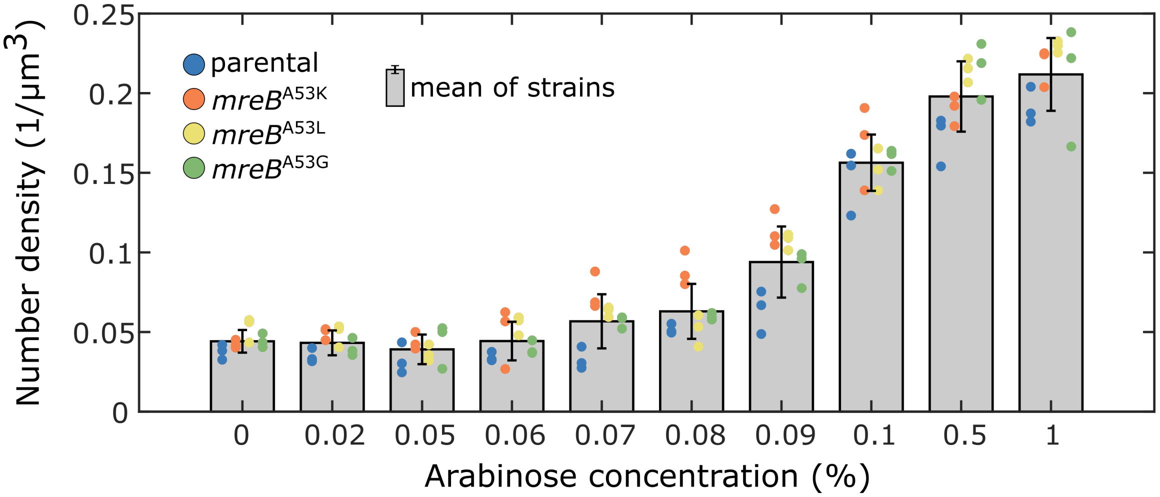

Fig H: The cell number density in *V. cholerae* biofilms changes with increasing arabinose concentration, for strains with an arabinose-inducible RbmA production. For each of the four mutant strains with different aspect ratios and arabinose-inducible RmbA production, three replicates of biofilms were grown in different arabinose concentrations. The mean and standard deviation of all four strains combined are shown as grey bars and error bars (as in Fig 2), whereas circles represent the individual data points. Points are colored according to the strain. Source data is available at DOI: 10.5281/zenodo.7077624.

E. PROTEOMICS METHODS

Sample Preparation

For LC-MS proteomics analysis, *V. cholerae* strains carrying a plasmid with a gentamicin resistance and an arabinose-inducible *rbmA* expression construct were grown over night in LB medium supplemented with gentamicin (final concentration 30 μg mL^{-1}) at 28 °C under shaking conditions. After overnight incubation, cells were back-diluted to an OD_{600} 0.05 in M9 medium supplemented with gentamycin (final concentration 30 μg mL^{-1}) and further incubated at 28 °C under shaking conditions. When the cultures reached OD_{600} 0.45, *rbmA* expression was induced by the addition of arabinose: 0.08% arabinose was used to compare the production of RbmA between *V. cholerae* strains with different *mreB* alleles (kdv611, kdv1232, kdv1291, kdv1403). Arabinose concentrations between 0 and 0.5% were used to investigate the relationship between the exogenous arabinose concentration and RbmA production in *V. cholerae* (strain kdv1082). After 4 h of incubation at 28 °C, 2.5×10^8 cells were collected, washed three times in PBS and lysed in 50 μL lysis buffer (2% sodium deoxycholate, 0.1 M TRIS, 10 mM TCEP, pH = 8.5) using strong ultra-sonication (10 cycles, Bioruptor, Diagnode). The protein concentration was determined by tryptophan fluorescence analysis [16] using a small sample aliquot. Sample aliquots containing 50 μg of total proteins were reduced for 10 min at 95 °C and alkylated at 15 mM chloroacetamide for 30 min at 37 °C. After diluting samples 1:1 (v:v) using 0.1 M TRIS (pH = 8.5), proteins were digested by incubation with sequencing-grade modified trypsin (1/50, w/w; Promega, Madison, Wisconsin) overnight at 37 °C. Then, the peptides were cleaned up using iST cartridges (PreOmics, Munich) according to the manufacturer's instructions. Samples were dried under vacuum and stored at -80 °C until further use.

LC-MS Analysis

Dried peptide samples were dissolved in aqueous 0.1% formic acid solution and 1 μg of peptides were LC-MS analyzed as described previously [17]. Chromatographic separation of peptides was carried out using an Ultimate 3000 nano-LC (Thermo Fisher Scientific), equipped with a heated RP-HPLC column (75 μm x 30 cm) packed in-house with 1.9 μm C18 resin (Reprosil-AQ Pur, Dr. Maisch). Peptides were analyzed per LC-MS/MS run using a linear gradient ranging from 95% solvent A (0.15% formic acid, 2% acetonitrile) and 5% solvent B (98% acetonitrile, 2% water, 0.15% formic acid) to 30% solvent B over 60 minutes at a flow rate of 300 nL/min. Mass spectrometry analysis was performed on Q-Exactive HF mass spectrometer equipped with a nanoelectrospray ion source (both Thermo Fisher Scientific) using data-independent acquisition. For MS1, 3e6 ions were accumulated in the Orbitrap cell over a maximum time of 100 ms and scanned at a resolution of 120,000 FWHM (at 200 m/z). MS2 scans were acquired at a target setting of 3e6 ions, accumulation time was set to auto and resolution to 30,000 FWHM (at 200 m/z). 30 MS2 windows of 18 m/z covering a mass range from 399.5 to 910.5 m/z with 1 m/z overall were collected per MS cycle. The normalized collision energy was set to 28% and all data were acquired in centroid mode.

The acquired raw-files were searched against a protein database containing sequences of the predicted Uniprot entries of *V. cholerae* N16961 (www.ebi.ac.uk, release date 2022/5/12) and commonly observed contaminants (in total 3,782 sequences) using the SpectroMine software (Biognosys, version 1.0.20235.13.16424) using default settings. In brief, the precursor ion tolerance was set to 10 ppm and fragment ion tolerance was set to 0.02 Da. The search criteria were set as follows: full tryptic specificity was required (cleavage after lysine or arginine residues unless followed by proline), 3 missed cleavages were allowed, carbamidomethylation (C) was set as fixed modification and oxidation (M) and N-terminal acetlyation as a variable modification. The false identification rate was set to 1%. For quantification, the raw files were loaded into SpectroNaut (Biognosys, v15) and searched against the spectral library generated by SpectroMine. The quantitative data were exported from SpectroNaut and statically analyzed using MSstats (v.4.1.3) [18].

F. INDIVIDUAL-BASED MODEL AND SIMULATIONS

Model description

Our single cell model is based on the agent-based framework described in [19], with modifications to include effective anisotropic friction and cell-floor attachment. Cells are modeled as ellipsoids of half-length l_α and half-width r_α. Each cell is described by its position $\hat{\boldsymbol{x}}_\alpha$, orientation $\hat{\boldsymbol{n}}_\alpha$ and effective local viscosity μ_α. For simplicity, we assume that all the cells have $r = 0.3422$ µm as their half-width and $\mu_m = 1$ Pa·s as their effective local viscosity [1], as recorded in Table E. The dynamics of the cells are approximated as over-damped, as cells live at low Reynolds number $Re \approx 10^{-4}$ [1]. Denoting the identity matrix by $\mathbf{I}$, the over-damped translational and orientational dynamics for a single cell are

$$\begin{aligned}\frac{\mathrm{d}\hat{\boldsymbol{x}}_\alpha}{\mathrm{d}t} &= \mathbf{\Gamma}^{-1}\left(-\frac{\partial U_{\text{bdy}}}{\partial \hat{\boldsymbol{x}}_\alpha} - \frac{\partial V_\alpha}{\partial \boldsymbol{x}_\alpha} - \frac{\partial U_{s,\text{cell}}}{\partial \hat{\boldsymbol{x}}_\alpha} - \frac{\partial U_{s,\text{bdy}}}{\partial \boldsymbol{x}_\alpha}\right)\\ \frac{\mathrm{d}\hat{\boldsymbol{n}}_\alpha}{\mathrm{d}t} &= \left(\mathbf{I} - \hat{\boldsymbol{n}}_\alpha \hat{\boldsymbol{n}}_\alpha^T\right)\left[\mathbf{\Omega}^{-1}\left(-\frac{\partial U_{\text{bdy}}}{\partial \hat{\boldsymbol{n}}_\alpha} - \frac{\partial V_\alpha}{\partial \hat{\boldsymbol{n}}_\alpha} - \frac{\partial U_{s,\text{cell}}}{\partial \hat{\boldsymbol{n}}_\alpha} - \frac{\partial U_{s,\text{bdy}}}{\partial \hat{\boldsymbol{n}}_\alpha}\right)\right]\end{aligned} \tag{S9}$$

where $\mathbf{\Gamma}$, $\mathbf{\Omega}$ are

$$\begin{aligned}\mathbf{\Gamma} &= \gamma_\alpha\left[\gamma_{\|}\left(\hat{\boldsymbol{n}}_\alpha \hat{\boldsymbol{n}}_\alpha^T\right) + \phi\gamma_\perp\left(\mathbf{I} - \hat{\boldsymbol{n}}_\alpha \hat{\boldsymbol{n}}_\alpha^T\right)\right]\\ \mathbf{\Omega} &= \omega_\alpha \omega_R \mathbf{I}.\end{aligned} \tag{S10}$$

Here, γ_α and ω_α are the typical translational and rotational drag coefficients for Stokes' drag for a spheroid ($\gamma_\alpha = 6\pi\mu_\alpha r_\alpha$, $\omega_\alpha = 8\pi\mu_\alpha l_\alpha r_\alpha^2$) [1, 19]. $\gamma_{\|}$, $\gamma_\perp$ and ω_R are dimensionless geometric parameters characterizing the longitudinal and transverse friction parameters that depend only on the aspect ratio $a = l_\alpha/r_\alpha$ of the cell, as given in [20]. ϕ is an anisotropic friction factor that suppresses transverse motions when ϕ increases ($\phi = 1$ for viscous fluid). We choose $\phi = 100$ as cells are moving in a matrix polymer network that grows around the cell, which can greatly suppress the transverse motions of the cell [21, 22].

Part of the interaction between a cell and the wall boundary is modeled with a repulsive-attractive interaction potential U_{bdy}, which is is proportional to the overlap between a cell and the wall boundary. The wall boundary is represented as a plane define by normal vector $\hat{\boldsymbol{N}}$ and a point on this plane $\boldsymbol{S}$. To determine this overlap, an overlap coordinate $z_o = l|\hat{\boldsymbol{n}}_\alpha \cdot \hat{\boldsymbol{N}}| + r - \hat{\boldsymbol{N}} \cdot (\hat{\boldsymbol{x}}_\alpha - \boldsymbol{S})$ is defined such that $z_o < 0$ implies no contact with the boundary and $z_o \geq 0$ implies contact with the boundary. We choose $\hat{\boldsymbol{N}} = [0, 0, 1]$ and $\boldsymbol{S} = [0, 0, 0]$ such that the wall is the xy-plane that crosses the origin. The repulsive-attractive interaction from the wall can be represented by

$$U_{\text{bdy}} = \begin{cases}\epsilon_{\text{bdy},a}\exp\left(-\frac{z_o^2}{\sigma_{\text{bdy},a}^2}\right) + \left(\epsilon_{\text{bdy},r} - \epsilon_{\text{bdy},a}\right), & z_o < 0\\ \epsilon_{\text{bdy},r}\exp\left(\frac{z_o}{\sigma_{\text{bdy},r}}\right), & z_o \geq 0\end{cases} \tag{S11}$$

where $\epsilon_{\text{bdy},a}$ captures the magnitude of the cell-boundary attraction, $\sigma_{\text{bdy},a}$ captures the range of the interaction, $\epsilon_{\text{bdy},r}$ captures the magnitude of the cell-boundary repulsion, and $\sigma_{\text{bdy},r}$ captures the range of the repulsion. The values of these parameters are listed in Table E.

The pairwise cell-cell interactions are described by two potentials V_α and $U_{s,\text{cell}}$, where V_α is the general pairwise cell-cell interaction that applies to all pairs of cells [1], and $U_{s,\text{cell}}$ is the polar attachment that only exists between two sibling cells after division or between the cell and the boundary. More specifically, V_α is the total potential for all $N-1$ pairwise cell-cell interactions between cell α and other $N-1$ cells β ($V_\alpha = \sum_{\beta=1,\beta\neq\alpha}^{N} U_{\alpha\beta}$). The interaction between cell α and cell β is governed by the cell-cell interaction potential $U_{\alpha\beta}$

$$U_{\alpha\beta} = \epsilon_0\epsilon_1\left[\exp\left(-\frac{\rho_{\alpha\beta}^2}{\lambda_r^2}\right) + \frac{\nu}{1 + \exp\left(\frac{\rho_a - \rho_{\alpha\beta}}{\lambda_a}\right)}\right]. \tag{S12}$$

Here $\rho_{\alpha\beta}$ is the normalized cell-cell distance in [1]. The strength of $U_{\alpha\beta}$ is described by ϵ_0 and adjusted by the shape factor $\epsilon_1(l_\alpha, l_\beta, r_\alpha, r_\beta, \hat{\boldsymbol{n}}_\alpha, \hat{\boldsymbol{n}}_\beta)$, depending on the relative cell orientations and cell shapes. λ_r describes the range of the osmotic cell-cell repulsion, ν describes the relative strength of cell-cell attraction, ρ_a describes the attraction position, and λ_a describes the width of the cell-cell attraction [1]. The value of ϵ_0 is listed in Table E. For the values of other interaction parameters, see **Simulation Parameters**.

The polar cell-cell attachment $U_{s,\text{cell}}$ after cell division is modeled as a harmonic spring between two sibling cells with spring constant k_{cell} and natural length r_{cell} [19]. As soon as a cell divides, a spring is assigned between the two closest endpoints of the daughter cells, with the spring potential

$$U_{s,\text{cell}} = k_{\text{cell}} \left(r_{\text{end}} - r_{\text{cell}} \right)^2 , \tag{S13}$$

where r_{end} is the distance between the two closest endpoints. The spring breaks at $0.5 \cdot \tau_g$ after division, where τ_g is the average doubling time measured from single-cell experimental data using the same technique as [1]. The values of k_{cell}, r_{cell} and τ_g are listed in Table E.

Pili-boundary attachment is modeled as a harmonic spring between a endpoint of cells and a attachment point on the floor with spring constant k_{bdy} and natural length r_{bdy}, to account for the elastic properties observed for type IV pili [23]. When the end point of a cell is close enough to the floor ($\leq 5 \cdot r_{\text{bdy}}$), a spring is assigned between the endpoint and the point on the floor that is closest to the endpoint, with a spring potential

$$U_{s,\text{bdy}} = k_{\text{bdy}} \left(r_{\text{cell-bdy}} - r_{\text{bdy}} \right)^2 , \tag{S14}$$

where $r_{\text{cell-bdy}}$ is the distance between the endpoint of cells and the attachment point on the floor. The spring breaks when its length is longer than $5 \cdot r_{\text{bdy}}$. When cells with cell-boundary pili divides, the daughter cells will inherit the cell-boundary pili if they inherit the endpoints that the pili are attached to. The values of k_{bdy} and r_{bdy} are listed in Table E.

The instantaneous cell length growth follows the growth equation in [1]

$$\frac{\mathrm{d}l_\alpha}{\mathrm{d}t} = \frac{l_\alpha}{\tau_g} \ln(2), \tag{S15}$$

where l_α is the instantaneous half-length of the cell and τ_g is the average doubling time. Cell widths are constant throughout simulations. A cell divides when it grows an additional length Δl from its birth length, where Δl is drawn from a Gaussian distribution with mean l_{add} and standard division σ_{add}. Cells divide into two daughter cells that have half the length of their parent cells and the same orientation as their parent cell. The value of l_{add} varies to account for difference *V. cholerae* mutants used in our experiments, and the value of σ_{add} is list in Table E.

Model implementation

A custom, highly parallelized individual cell-based code employing graphics processing units (GPUs) was developed to perform the simulations based on [1, 19]. At each time step, cell-cell interactions between all pairs of cells and cell-floor interaction of all the cells are evaluated. A standard explicit Euler scheme is used to numerically integrate (S9) and (S15) in non-dimensional form, with $r = 0.3422\,\mu\text{m}$ as the length scale, the translational time $\tau_t = 5.65$ s as the time scale [1] and $\epsilon = 5 \cdot 10^{-20}$ J [1] as the energy scale.

Simulation parameters

To determine the values of interaction parameters in this model for different mutant and difference arabinose concentration level, a systematic scan of division length and four cell-cell interaction parameters (width of cell-cell repulsion λ_r, width of cell-cell attraction λ_a, position of cell-cell attraction ρ_a and strength of cell-cell attraction ν) was performed, resulting 6825 biofilm simulations. For a combination of mutant and arabinose concentration level in experiments, the best-fit simulation is obtained by choosing smallest average Cd to the corresponding experimental biofilms found within the parameter space searched. To restrict the size of the parameter space searched, we only searched over simulations which have an average aspect ratio at most 0.6 away from the experimental biofilm. For combinations of four different mutants and six different arabinose concentration, the renderings of the best-fit simulations are shown in Fig 3 in the main text and their corresponding interaction parameters are plotted in Fig I. The average and the standard deviation of Cd between the corresponding experiments ($n = 3$) and the best-fit simulation are plotted in Fig J. Other simulations parameters are determined from previous single-cell biofilm experiments [1, 19] and recorded in Table E.

Parameter	Value	Unit	Description
r	0.3422	µm	Average half-width of the bacteria, obtained from experimental measurements of *V. cholerae* biofilms.
τ_g	6130	s	Growth time constant [1].
μ_m	1	Pa·s	Estimate of the dynamic viscosity of EPS matrix at room temperature [1].
$\epsilon^*_{\mathrm{bdy},r}$	10		Ratio comparing the strength of bacteria-boundary repulsion to the strength of the bacteria-bacteria interaction ($\epsilon^*_{\mathrm{bdy},r} = \epsilon_{\mathrm{bdy},r}/\epsilon_0$) [1].
$\sigma_{\mathrm{bdy},r}$	0.3422	µm	Non-dimensional boundary repulsion length scale parameter.
$\epsilon^*_{\mathrm{bdy},a}$	0.5		Ratio comparing the strength of bacteria-boundary attraction to the strength of the bacteria-bacteria interaction ($\epsilon^*_{\mathrm{bdy},a} = \epsilon_{\mathrm{bdy},a}/\epsilon_0$).
$\sigma_{\mathrm{bdy},a}$	0.3422	µm	Non-dimensional boundary repulsion length scale parameter.
τ_t	5.65	s	Translational time scale due to repulsion in matrix (typical time needed for daughter cells in matrix to reach their equilibrium configurations due to repulsion after cell division) [1].
σ_{add}	0.08	µm	Standard deviation of length added to bacteria after division to compute division length [19].
ϵ_0	$5{\cdot}10^{-20}$	J	Strength of the osmotic pressure-mediated cell-cell repulsion.
k_{cell}	$8.5{\cdot}10^{-20}$	J·µm^{-2}	Spring constant for the additional directional cell-cell attraction.
r_{cell}	0.3422	µm	Natural length of the spring between sibling cells.
k_{bdy}	$8.5{\cdot}10^{-20}$	J·µm^{-2}	Spring constant for the additional directional cell-cell attraction.
r_{bdy}	0.3422	µm	Natural length of the spring between sibling cells.

Table E: Key simulation parameters in individual-based simulations.

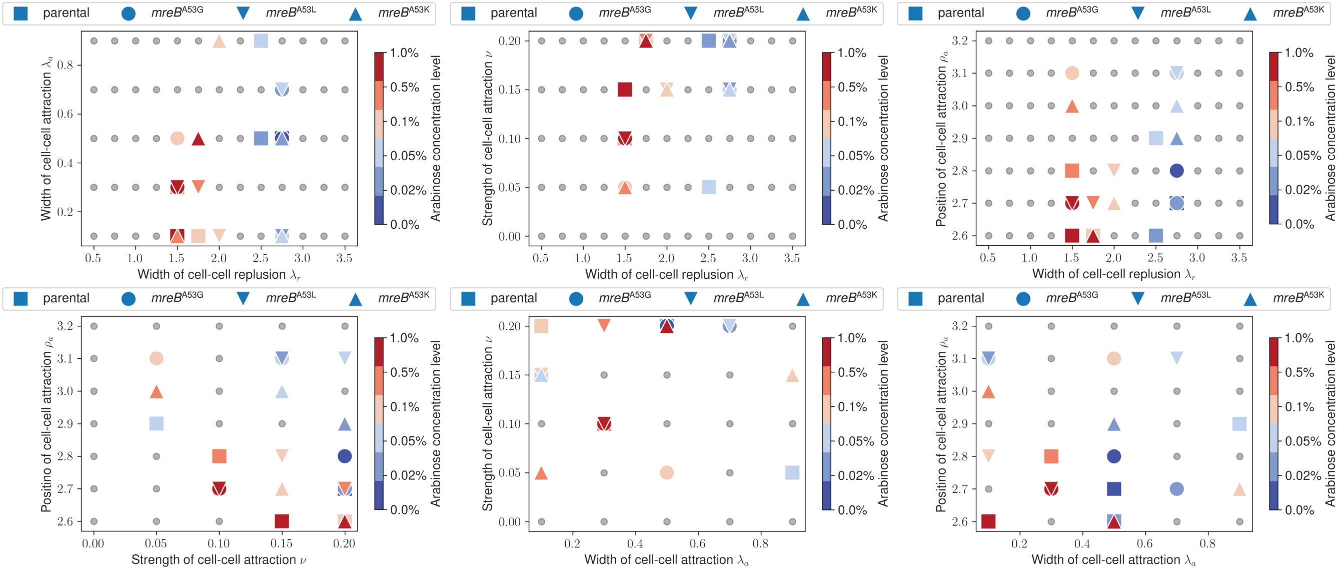

Fig I: Cell-cell interaction parameters of the best-fit simulations in the main text Fig 3. Source data is available at DOI: 10.5281/zenodo.7077624.

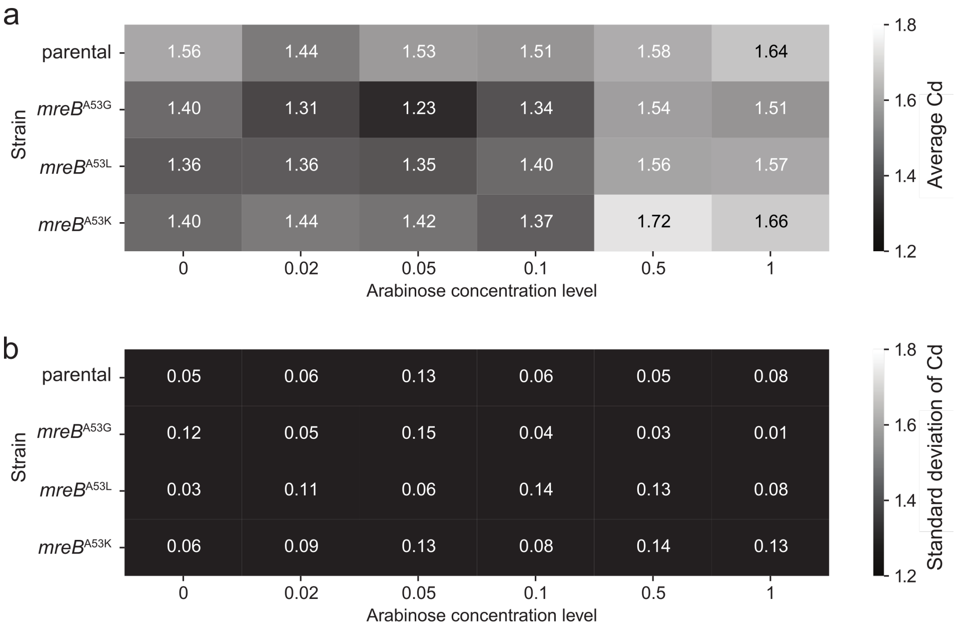

Fig J: Average (a) and standard deviation (b) of Cd between the experiments ($n = 3$) and the best-fit simulation in the main text Fig 3. Source data is available at DOI: 10.5281/zenodo.7077624.

[1] R. Hartmann, P. K. Singh, P. Pearce, R. Mok, B. Song, F. Díaz-Pascual, J. Dunkel, and K. Drescher, Nat. Phys. **15**, 251 (2019).
[2] K. L. Meibom, X. B. Li, A. T. Nielsen, C.-Y. Wu, S. Roseman, and G. K. Schoolnik, Proc. Natl Acad. Sci. U.S.A. **101**, 2524 (2004).
[3] K. Drescher, J. Dunkel, C. D. Nadell, S. Van Teeffelen, I. Grnja, N. S. Wingreen, H. A. Stone, and B. L. Bassler, Proc. Natl Acad. Sci. U.S.A. **113**, E2066 (2016).
[4] R. D. Monds, T. K. Lee, A. Colavin, T. Ursell, S. Quan, T. F. Cooper, and K. C. Huang, Cell Rep. **9**, 1528 (2014).
[5] D. O. Serra, A. M. Richter, G. Klauck, F. Mika, and R. Hengge, mBio **4** (2013), 10.1128/mBio.00103-13.
[6] N. Grantcharova, V. Peters, C. Monteiro, K. Zakikhany, and U. Römling, J. Bacteriol. **192**, 456 (2010).
[7] L. Vidakovic, P. K. Singh, R. Hartmann, C. D. Nadell, and K. Drescher, Nat. Microbiol. **3**, 26 (2017).
[8] V. De Lorenzo and K. N. Timmis, Meth. Enzymol. **235**, 386 (1994).
[9] U. Römling, W. D. Sierralta, K. Eriksson, and S. Normark, Mol. Microbiol. **28**, 249 (1998).
[10] J. G. Malone, T. Jaeger, C. Spangler, D. Ritz, A. Spang, C. Arrieumerlou, V. Kaever, R. Landmann, and U. Jenal, PLoS Pathog **6**, e1000804 (2010).
[11] K. Skorupski and R. K. Taylor, Gene **169**, 47 (1996).
[12] K. Drescher, C. D. Nadell, H. A. Stone, N. S. Wingreen, and B. L. Bassler, Current Biology **24**, 50 (2014).
[13] C. D. Nadell, K. Drescher, N. S. Wingreen, and B. L. Bassler, ISME J. **9**, 1700 (2015).
[14] P. K. Singh, S. Bartalomej, R. Hartmann, H. Jeckel, L. Vidakovic, C. D. Nadell, and K. Drescher, Curr. Biol. **27**, 3359 (2017).
[15] R. Hartmann, H. Jeckel, E. Jelli, P. K. Singh, S. Vaidya, M. Bayer, D. K. H. Rode, L. Vidakovic, F. Díaz-Pascual, J. C. N. Fong, A. Dragos, O. Lamprecht, J. G. Thöming, N. Netter, S. Haussler, C. D. Nadell, V. Sourjik, A. T. Kovacs, F. H. Yildiz, and K. Drescher, Nat. Microbiol. **6**, 151 (2021).
[16] J. R. Wiśniewski and F. Z. Gaugaz, Analytical Chemistry **87**, 4110 (2015).
[17] E. Ahrné, T. Glatter, C. Viganò, C. v. Schubert, E. A. Nigg, and A. Schmidt, Journal of Proteome Research **15**, 2537 (2016).
[18] M. Choi, C.-Y. Chang, T. Clough, D. Broudy, T. Killeen, B. MacLean, and O. Vitek, Bioinformatics **30**, 2524 (2014).
[19] P. Pearce, B. Song, D. J. Skinner, R. Mok, R. Hartmann, P. K. Singh, H. Jeckel, J. S. Oishi, K. Drescher, and J. Dunkel, Phys. Rev. Lett. **123**, 258101 (2019).
[20] Y. Han, A. Alsayed, M. Nobili, and A. G. Yodh, Phys. Rev. E **80**, 011403 (2009).
[21] T. Munk, F. Höfling, E. Frey, and T. Franosch, Euro. Phys. Lett. **85**, 30003 (2009).
[22] F. Höfling, E. Frey, and T. Franosch, Phys. Rev. Lett. **101**, 120605 (2008).
[23] L. Craig, K. T. Forest, and B. Maier, Nat. Rev. Microbiol. **17**, 429 (2019).
